# Exploring Naturally Tailored Bacterial Outer Membrane Vesicles for Selective Bacteriostatic Implant Coatings

**DOI:** 10.1002/advs.202405764

**Published:** 2024-08-21

**Authors:** Zilin Zhou, Lizhong Sun, Yuanyuan Tu, Yingming Yang, Ailin Hou, Jiyao Li, Jun Luo, Lei Cheng, Jianshu Li, Kunneng Liang, Jiaojiao Yang

**Affiliations:** ^1^ State Key Laboratory of Oral Diseases National Clinical Research Center for Oral Diseases West China Hospital of Stomatology Sichuan University Chengdu 610041 P. R. China; ^2^ Department of Cariology and Endodontics West China Hospital of Stomatology Sichuan University Chengdu 610041 P. R. China; ^3^ Department of Jinjiang Outpatient West China Hospital of Stomatology Sichuan University Chengdu 610041 P. R. China; ^4^ College of Polymer Science and Engineering State Key Laboratory of Polymer Materials Engineering Sichuan University Chengdu 610065 P. R. China; ^5^ Med‐X Center for Materials Sichuan University Chengdu 610065 P. R. China

**Keywords:** bacterial communication, interface modification, outer membrane vesicles, selective bacterial inhibition

## Abstract

In treating infectious diseases, achieving selective bacterial inhibition is crucial for preserving the microecological equilibrium. The current approaches predominantly rely on synthetic materials tailored to specific bacteria, considering their cell walls or oxygen requirements. Herein, inspired by intricate bacterial communication, a natural implant is proposed coating utilizing bacterial outer membrane vesicles (OMVs), essential components in bacterial signaling, integrated onto diverse implant surfaces through a universal poly (tannic acid) bridging layer. This coating is homogenous and stable, unexpectedly promoting the proliferation of parental bacteria while inhibiting heterologous bacteria both in vitro and in vivo. Through high‐throughput sequencing and bioinformatics analysis, the selective bacteriostatic ability arises from OMVs, upregulating anti‐oxidative stress genes in heterologous bacteria and activating biofilm‐related genes in parental bacteria. This study positions OMVs as an appealing biomaterial for selective bacterial inhibition through a biological approach, showcasing their potential in regulating the microecological balance through a natural interface modification strategy.

## Graphical abstract

Bacterial outer membrane vesicles (OMVs) are now used to construct implant coatings, showing unexpected selective bacteriostasis at the macroscopic level. Using a simple polyphenol bridging technology stabilizes bacterial OMVs on various implant surfaces. This OMVs coating gives implants the ability to selectively inhibit bacteria. This method provides natural alternatives for potential selective antimicrobial interventions in treating infectious diseases.

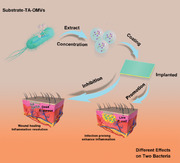

## 1 Introduction

Bacterial infections pose a significant threat to human health,^[^ ^1^, ^2^ ^]^ exacerbated by the overuse and abuse of antibiotics.^[^ ^3^, ^4^ ^]^ While alternative antibacterial therapies, including photothermal therapy (PTT), photodynamic therapy (PDT), and quaternary ammonium salt (QAS),^[^ ^5^ ^]^ have been reported, their broad‐spectrum antibacterial nature engenders a risk of disrupting microecological balance, leading to adverse effects on the body, such as inflammation and immune dysregulation.^[^ ^6^, ^7^ ^]^ Therefore, there is a pressing need to develop a reliable therapy that selectively inhibits harmful bacteria while preserving health‐associated bacteria to ensure microecological balance in treating bacterial infections.

Existing selective antibacterial agents, predominantly synthetic materials, determine their antibacterial spectrum based on the structure of bacteria (e.g., the molecular composition of their cell walls and their metabolic pathways).^[^ ^8^, ^9^ ^]^ For instance, β‐lactam antibiotics exhibit remarkable specificity against Gram‐positive bacteria by inactivating β‐lactamases, while Gram‐negative bacteria remain unaffected due to differences in their cell wall composition. Targeting bacterial metabolic pathways can also exert selective antibacterial effects. For instance, Yang et al. have successfully engineered supramolecular complexes that generate free radicals when triggered by near‐infrared irradiation.^[^ ^10^ ^]^ Bacteria with varying reduction capacities toward these complexes determine their susceptibility to damage by the generated free radicals. However, despite these advances, current selective antibacterial strategies fall short in two aspects: i) Distinguishing between beneficial and harmful bacteria solely based on cell wall structure or sensitivity to oxygen is not feasible, and ii) strategies that completely eliminate one specific type or class of bacteria may disrupt the microecological balance composed of various bacteria working together.

In bacterial microbiota, dominant strains often exhibit higher population densities compared to other bacteria.^[^ ^11^, ^12^ ^]^ As dominant bacteria proliferate, the population of non‐dominant bacteria tends to decrease due to factors such as changes in the growth environment.^[^ ^13^ ^]^ Research indicates that information exchange among bacteria, as well as between bacteria and the host, plays a crucial role in the formation of dominant strains, facilitating inter‐species bacterial interactions aimed at “seeking benefits and avoiding harm.” Outer membrane vesicles (OMVs)^[^ ^14^, ^15^ ^]^ are abundant in bacteria,^[^ ^16^ ^]^ playing a vital role in this signal communication among bacteria.^[^ ^17^, ^18^ ^]^

This study, for the first time, introduces bacterial OMVs as a naturally derived biomaterial into a proof‐of‐concept implant coating for selective bacterial inhibition, specifically using OMVs extracted from *Escherichia coli* (*E. coli)* as the model (Figure S1A, Supporting Information). Tannic acid (TA), a plant polyphenol with high catechol/pyrogallol content,^[^ ^19^, ^20^ ^]^ forms a poly (tannic acid) layer on various implant surfaces, acting as an ideal intermediate to bridge OMVs and implant surfaces. This creates a stable and integrated biofunctional implant coating based on OMVs, eliminating the necessity for synthetic materials. This connection structure is simple and easy to operate and enhances the stability of OMVs. The substrate and TA are mainly connected by coordination bonds (Ti and Hap) and hydrogen bonds (Si), and the TA and OMVs are mainly connected by hydrogen bonds and Schiff base reaction between catechol groups of TA and proteins on the surface of OMVs.^[^ ^21^ ^]^ This OMV coating efficiently inhibits heterogenous microorganisms in vitro, while notably enhancing the growth of parent bacteria. By analyzing the structure of disrupted OMVs and conducting high‐throughput sequencing and bioinformatics analysis, we discover that OMVs exert distinct regulatory effects on the energy metabolism, respiratory chain, cellular transport processes, and antioxidant levels of different bacteria. This results in the upregulation of anti‐oxidative stress genes in heterozygous bacteria and the activation of biofilm‐related genes in parental bacteria. This reports the mechanism of selective antimicrobial inhibition of OMVs from the perspective of genetic alterations. Notably, the OMVs coating facilitates wound healing in response to heterologous bacterial infection, while exacerbating infections in parental bacterial wounds in vivo. This phenomenon, where the coating has opposite effects on different bacteria, is referred to as the “angel and devil” effect (Figure S1B, Supporting Information). As a result, OMVs demonstrate significant potential for selective bacterial inhibition with high precision rather than the common sense of controlling bacteria. Moreover, this natural and mild interface modification strategy holds promise for regulating microecological balance in infectious disease treatment.

## 2 Results and Discussion

### 2.1 Characterization of OMVs

**Figure 1:**
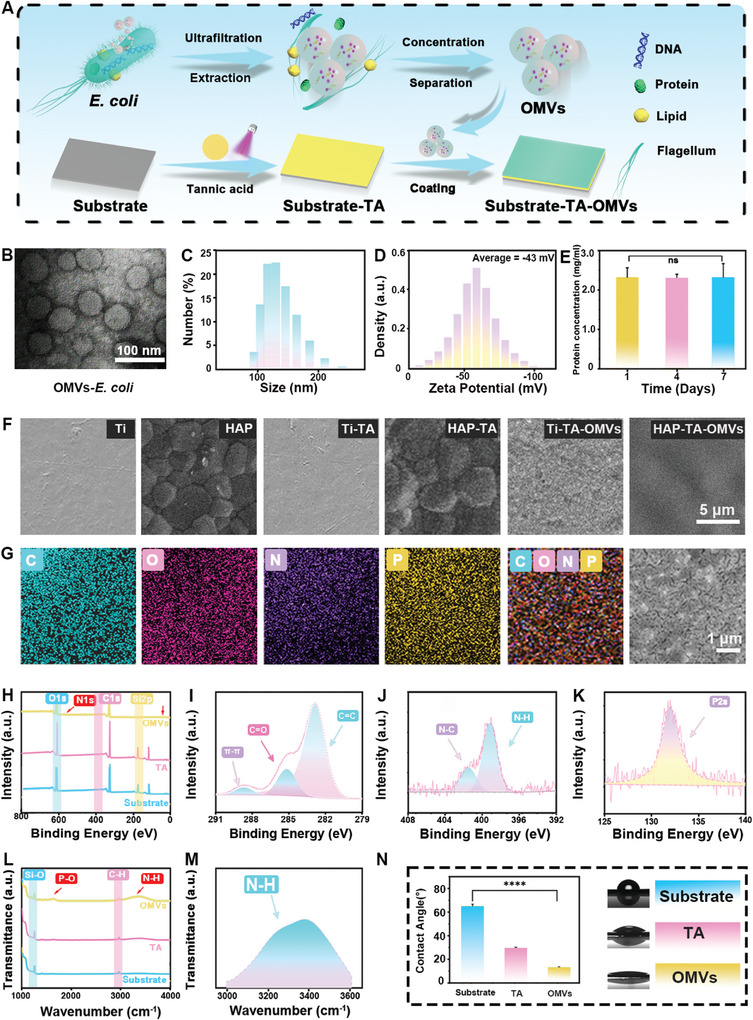
Characterization of OMVs and OMVs coating. A) The process involves extracting OMVs and constructing a Substrate‐TA‐OMVs system. B) TEM image, C) dynamic size, and D) zeta potential of OMVs. E) Bicinchoninic Acid Assay (BCA) of OMVs on days 1–7. F) SEM image, G) elemental mapping, H) XPS spectra of Substrate, TA, and OMVs. Core level spectra of I) C 1s of OMVs, J) N 1s of OMVs, and K) P 2p of OMVs. L) FTIR spectra of Substrate, TA, and OMVs. Core functional group of M) N─H of OMVs. N) WCA of Substrate, TA, and OMVs. Data are presented as mean ± SD (*n* = 3) and analyzed using a one‐way ANOVA, **p < 0.01, ***p < 0.001, ns, no significance.

First, we prepared OMVs from *E. coli*. The morphology of representative OMVs was evaluated by transmission electron microscope (TEM) (**Figure** **1**B), revealing a spherical morphology. The presence of multiple vesicles within the structure confirmed the efficacy of the extraction process.^[^ ^22^, ^23^, ^24^ ^]^ The mean diameter was determined to be 119.92 nm through dynamic light scattering (DLS) measurements (Figure 1C). This is consistent with the observed size range of OMVs, which falls within the 20–200 nm range as previously reported before.^[^ ^25^, ^26^ ^]^ All vesicles exhibited a negative surface charge (ζ potential) of −43.15 mV (Figure 1D). This indicates that OMVs have good stability and strong electrostatic repulsion between particles. Furthermore, the protein concentration of the OMVs suspension remains constant over the course of 1, 4, and 7 d of storage, indicating that the OMVs themselves possess inherent robust stability (Figure 1E).

### 2.2 Fabrication of the Poly (Tannic Acid)‐Assisted OMVs Coating

The fabrication process of the poly(tannic acid)‐assisted OMVs coating underwent evaluation through Scanning Electron Microscop (SEM), X‐ray Photoelectron Spectroscopy (XPS), Fourier Transform Infrared Spectrometer (FTIR), Water Contact Angle (WCA) measurements, and Atomic Force Microscope (AFM). Three distinct structures are constructed utilizing silicone sheet, hydroxyapatite (HAP) sheet, and titanium sheet substrates to assess the adherence of OMVs to these materials. “Substrate‐TA” is abbreviated as “TA”, and “Substrate‐TA‐OMVs” is abbreviated as “OMVs”. SEM observation reveals numerous uniform spheroidal particles on the surface of the substrates in the OMVs group (Figure 1F; Figure S2, Supporting Information). Notably, no significant difference is observed between the Substrate and TA groups. The energy dispersive spectrometer (EDS) elemental mapping images in the OMVs group demonstrate a homogeneous distribution of typical elements (Figure 1G). Furthermore, AFM observations of the surface morphology show surface roughness parameters (Ra) of 1.81 ± 0.14 nm, 1.62 ± 0.21 nm, and 3.91 ± 1.45 nm for the Substrat, TA, and OMVs groups, respectively (Figure S3, Supporting Information). We speculate that the increase of Ra in group OMVs is attributed to the attachment of OMVs, which is in accordance with the SEM results.

As presented in Figure 1H, XPS was employed to detect the elemental changes on material surfaces. It is discovered that, compared to the Substrate group, the TA group has significantly decreased levels of Si and increased levels of C and O. This transformation is attributed to the TA deposition. In group OMVs, the increase in N content and the appearance of P at 130.5 eV confirm the successful attachment of OMV, which aligns with EDS findings in Figure 1G. As shown in Figure 1I, the TA group showcases a C═C peak of 283.80 eV, a C═O peak of 286.96 eV, as well as a π─π bond peak at 290.40 eV, which further proves the deposition of TA on the substrates. Meanwhile, peaks at 398.78 eV (N─H), 400.28 eV (N─C), and 134.38 eV (P─O) are attributed to the attachment of OMVs (Figure 1J,K). This is similarly confirmed by the titanium‐based OMVs group (Figure S4, Supporting Information). Additionally, FTIR was employed to investigate the surface chemical structure information. Compared with the TA group, PO^2‐^ stretching movement of phospholipids at 1065 cm^−1^ (Figure 1L)^,^ and N─H stretching vibrations at 3380 cm^−1^ (Figure 1M) are found in group OMVs. These peaks are only observed in OMVs and are related to OMVs deposition. This is similarly confirmed by the titanium‐based OMVs group (Figure S5, Supporting Information). After applying the poly(tannic acid) coating on the Substrate (group TA) through TA (Figure 1N), it is observed that TA, with numerous carbonyl groups, significantly improves the surface hydrophilicity. Subsequently, after coating OMVs on the TA‐Substrate (group OMVs), the WCA further dropped down to 15.98 ± 0.45° due to the abundance of hydrophilic functional groups present in both TA and OMVs.

In the OMVs system, TA plays a crucial role as a biological cross‐linking agent capable of forming a poly (tannic acid) coating on a substrate, acting as a bridge to stably incorporate OMVs due to its high affinity for biocomponents.^[^ ^27^, ^28^ ^]^ SEM, AFM observations, and auxiliary chemical factor proof (XPS and FTIR) conclude that the coating of TA and OMVs has been successfully deposited on substrates.

### 2.3 In Vitro Selective Bacteriostatic Properties

**Figure 2:**
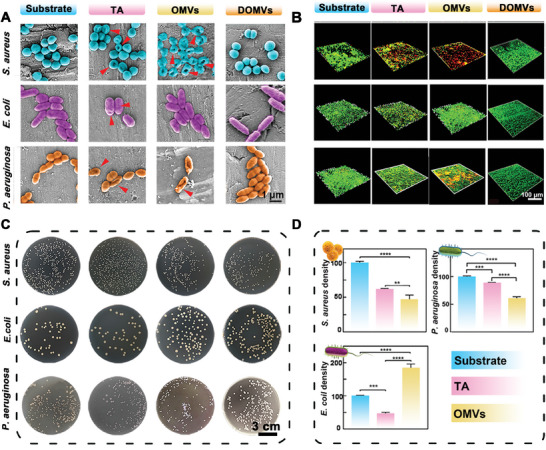
Selective bacteriostatic performances of OMVs and DOMVs coatings in vitro. A) SEM images of *S. aureus*, *E. coli*, and *P. aeruginosa* cultured with Substrate, TA, OMVs, and DOMVs. The red arrows reveal the contracted bacterial cytomembrane. B) LIVE/DEAD‐stained confocal images of *S. aureus*, *E. coli*, and *P. aeruginosa* treated with Substrate, TA OMVs, and DOMVs. C) Bacterial colonies treated with Substrate, TA OMVs, and DOMVs. D) Bacterial density of *S. aureus, E. coli*, and *P. aeruginosa* treated with Substrate, TA, and OMVs. Data are presented as mean ± SD (*n* = 3) and analyzed using a one‐way ANOVA, **p < 0.01, ***p < 0.001, ****p < 0. 0001, ns, no significance.

The in vitro bacteriostatic assays of OMVs were tested against homologous *E. coli* bacteria and various non‐homologous bacteria. These include gram‐positive bacteria (*Staphylococcus aureus* (*S. aureus*), *Staphylococcus epidermidis* (*S. epidermidis*) and *Enterococcus faecalis* (*E. faecalis*)) and gram‐negative bacteria (*E. coli* and *Pseudomonas aeruginosa* (*P. aeruginosa*)) (**Figure** **2A,B**; Figures S6–S8, Supporting Information). In vitro bacteriostatic assays, we opted for silicone sheets and titanium sheets as substrate. The survival ratios of *S. aureus* in the TA and OMVs to silicone substrate group are 47.28% ± 7.72% and 26.41% ± 5.27%, respectively (Figure S6, Supporting Information). In the TA and OMVs to titanium substrate group, the survival ratios are 61.83% ± 1.02% and 46.95% ± 6.44%, respectively (Figure 2D). While *P. aeruginosa*, *S. epidermidis*, and *E. faecalis* treated with OMVs all show a modest decrease in survival rate (Figure S8, Supporting Information), the survival rate of *E. coli* exhibits growth in the presence of OMVs relative to TA and Substrate group. The inhibitory effect on heterologous bacteria and the growth promotion for homologous bacteria observed in in vitro experiments highlight the selective inhibition effect of OMVs on different bacterial species.

Moreover, SEM provides a visual demonstration of similar results (Figure 2A; Figure S9, Supporting Information). *S. aureus*, *S. epidermidis*, *E. faecalis*, and *P. aeruginosa* all exhibit more severe cell membrane lesions and exudation of cell contents in the OMVs group that confirm the killing ability of OMVs. The parental *E. coli* in the Substrate, TA, and OMVs groups show smooth surfaces and typical shapes. Furthermore, more *E. coli* is detected in the OMVs group. To assess the selective bacteriostatic activity of OMVs, LIVE/DEAD staining experiments were conducted. The representative images are displayed in Figure 2B and Figures S10 and S11 (Supporting Information). Based on the fluorescence images of *S. aureus*, *E. faecalis*, *S. epidermidis*, and *P. aeruginosa*, the majority of live bacteria are present in the images of substrate and TA. Most of the dead bacteria are in the OMVs group. The bacteriostatic effect of the OMVs group is more pronounced than that of the TA group, indicating that the bacteriostatic effect does not come from the coverage of TA, but from the coverage of OMVs. This is also demonstrated by experiments performed on titanium substrates (Figures S11 and S12, Supporting Information). It is also surprising that the OMVs extracted from *E. coli* do not produce significant bacteriostatic activity against *E. coli* but produce the same relatively stable promoting effect as colony‐forming units (CFU) (Figure 2C). Although the effect of the coating mainly came from OMVs, the effect of the OMVs alone group (OMVs (−)) was not as good as that of the Substrate TA‐OMVs (OMV (+)) (Figures S13–S17, Supporting Information). Therefore, in vitro experiments have demonstrated that OMVs coatings can selectively kill bacteria and stimulate the growth of parental bacteria.

Although the material is not considered to be very strong against *P. aeruginosa*, the effect can still be boosted by other assists.^[^ ^29^, ^30^ ^]^ (Figures S15 and S18, Supporting Information) Precision antibiotics are indispensable in certain special treatment scenarios, as mentioned earlier. Unlike previously reported the common sense of control bacteria and the selective antibacterial agents,^[^ ^31^, ^32^ ^]^ our approach achieves a significantly higher level of precision in the bactericidal selection and results in a more stable promotion effect on the parent bacteria. This has never been reported before and is more consistent with a precise bacteriostatic treatment strategy. Furthermore, our study shows that the efficacy of the OMVs coating is not reliant on the structural role of the bacterial membrane or cell wall, nor is it influenced by external conditions such as oxygen levels.

The longevity of coating stability holds paramount importance. Therefore, an examination of morphological and biological stability was conducted over a period spanning 7 to 21 days. The OMVs were pre‐stained with DiD, and morphological variations were observed using fluorescence microscopy. The static stability of the OMVs on the substrate surfaces is depicted in Figure S19A (Supporting Information), where the fluorescence of distinct spots is visualized on the 3D reconstruction map. Results reveal no significant change in fluorescence intensity quantification (Figure S19B,C, Supporting Information) over the 3 weeks. The surface morphology of the OMVs group exhibits no discernible vibration. To further assess stability, XPS and FTIR were employed to observe surface elements and functional groups at 1, 4, and 7 days. Figure S19D,E (Supporting Information) demonstrates no noteworthy reduction in surface N and P elements. These findings collectively corroborate the stability of the coating structure. For additional verification, we conducted an infrared analysis of the OMVs coating attached to HAP, revealing no significant alteration in groups (Figure S19F,G, Supporting Information). Moreover, to affirm the sustained biological functions of the OMVs coating, bacteriostatic testing was performed on days 1, 4, and 7. The results demonstrate that the interaction with bacteria remained consistently unaltered, aligning with the aforementioned stability assessments (Figure S19H–J, Supporting Information). These results suggest that in the TA‐bonded system, the deposition of OMVs on the substrate exhibits relative stability, demonstrating robust endurance in both morphology and biological effects.

To further investigate whether the precise selective bacteriostatic mechanism of OMVs is mediated by the vesicular contents or vesicular structure. We extracted the vesicle membranes of *E. coli* OMVs and tested them by DLS and TEM. The results indicate that the OMVs have been successfully destroyed in structure and are referred to as destroyed OMVs (DOMVs). Furthermore, by staining the prepared Substrate‐TA‐DOMVs with DiD, we confirmed that the DOMVs successfully covered the substrate (Figure S20, Supporting Information). SEM analysis reveals no significant difference in cell membrane integrity among different bacteria in the Substrate, TA, and DOMVs groups (Figure 2A; Figure S21, Supporting Information). Especially, the DOMVs group loses its significant bacteriostatic properties for heterologous bacteria. Additionally, the non‐bacteriostatic activity of DOMVs for the three bacteria in vitro is further confirmed by the spread plate method (Figure 2C). The bacteria density and survival rates of the three groups are not markedly different (Figure 2C; Figure S22, Supporting Information). Additionally, LIVE/DEAD‐stained images reveal that the number of living bacteria of *S. aureus*, *E. coli*, and *P. aeruginosa* is not significantly reduced. The intensity of the green fluorescence (live bacteria) is significantly higher than that of the red fluorescence (dead bacteria) in the DOMVs group (Figure 2B). In summary, the mere vesicular membranes alone are insufficient to maintain selective bacteriostatic activity. This implies that the primary factor contributing to the selective bacteriostatic efficacy is the cargo contained within the vesicles, and previous studies have demonstrated the crucial role of vesicle‐carried specific enzymes in bacterial communication and population competition.^[^ ^10^ ^]^ These enzymes can selectively eliminate foreign bacteria while protecting the host microbiota.

### 2.4 Transcriptomic Analysis of *S. aureus* Exposed to OMVs

The present study utilized RNA‐Seq to analyze the differentially expressed genes (DEGs) of *S. aureus*. We further explored the OMVs inhibit the growth of bacteria from the gene level change, this little has been reported. The credibility of the results is confirmed using ribosomal contamination assessment, principal component analysis, and sample clustering analysis. A total of 1181 DEGs (1017 upregulated and 164 downregulated) with highly significant expression patterns are identified and shown on heat maps(Figures S23–S26, Supporting Information).

**Figure 3:**
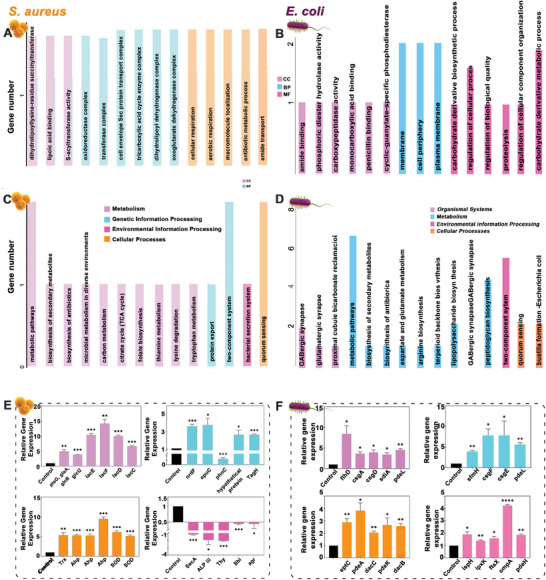
Changes in the transcriptome of *S. aureus* or *E. coli* were treated with OMVs coating. The gene ontology (GO) enrichment analysis of the gene functions of upregulated DEGs of *S. aureus* A) and *E. coli* B). GO enrichment analysis of the gene functions of downregulated DEGs in *S. aureus* C) and *E. coli* D). E) The DEGs in *S. aureus* involved in antioxidative stress, and cell membrane integrity related to intracellular and extracellular transport were evaluated. Factors associated with bacterial virulence were evaluated. F) The DEGs in *E. coli* involved in bacterial adhesion, and cell membrane integrity related to intracellular and extracellular transport, and were evaluated. Data are presented as mean ± SD (*n* = 3) and analyzed using a one‐way ANOVA, **p < 0.01, ***p < 0.001, ns, no significance.

GO enrichment analysis of the upregulated genes reveals that the DEGs associated with transporter activity, antioxidant function, and catalytic activity were stimulated by the OMVs (**Figure** **3**A). Additionally, the KEGG pathway enrichment analysis shows that genes related to oxidative phosphorylation, the TCA cycle, and metabolic pathways were upregulated, providing some insight into the observed changes in GO enrichment function (Figure 3C). Upregulated genes in the *Glc*, *Lac*, and *Fru* families linked to the PTS system^[^ ^33^, ^34^, ^35^, ^36^ ^]^ and genes like *Opu BC* and *Phn C/D/E* indicate changes in *S. aureus* transport function,^[^ ^37^ ^]^ affecting substance translocation and ABC transporter activity in response to external stimuli (Figure 3E).

GO enrichment analysis reveals upregulation of anti‐oxidative stress genes, including *TrxA/B*, *Ahp*, and *SOD* families (Figure 3E). This suggests increased production of reactive oxygen species and activation of the bacterial anti‐oxidative stress function. OMVs treatment induces *S. aureus* to generate excessive ROS. Downregulation of genes in oxidoreductase, *Sec* protein transport complex, and cellular respiration (Figure 3A) puts bacteria in a hypoxic state, enhancing ROS production and inducing apoptosis. This aligns with decreased virulence genes, like those in secretion systems and quorum sensing^[^ ^38^, ^39^ ^]^ (Figure 3C,E), confirming OMVs' inhibitory effect on *S. aureus*.

Our hypothesis is that the bacteriostatic mechanism of OMVs is linked to excessive ROS production, affecting bacterial transport and oxidative phosphorylation. This increases ROS levels, activating the anti‐oxidative stress system (*SOD*, *Trx*). Excessive ROS damages bacterial structures and respiratory function, leading to apoptosis. OMVs selectively inhibit non‐parental bacteria by disrupting cell membranes, transport systems, energy metabolism, and oxidative stress responses, causing direct damage to *S. aureus*.

### 2.5 Transcriptomic Analysis of *E. coli* Exposed to OMVs

In this section, a statistical analysis was conducted to compare the DEGs between the control group and the group treated with *E. coli* OMVs, which has little mentioned before. The credibility of the results was confirmed using ribosomal contamination assessment, principal component analysis, and sample clustering analysis. Changes in differential genes are also shown on the heat map. A total of 946 DEGs are identified, with 736 showing upregulation and 210 showing downregulation (Figures S27–S29, Supporting Information). Enrichment analysis using GO analysis reveals that the upregulated DEGs are associated with the regulation of biological adhesion (Figure 3B). Additionally, KEGG pathway analysis shows enrichment of biofilm formation among the upregulated gene (Figure S30, Supporting Information).

Studies show biofilms play a key role in bacterial resistance. While many bacteriostatic materials inhibit biofilm formation, OMVs may promote bacterial growth by upregulating biofilm‐related pathways. KEGG pathway ko02026 and genes like *FlhD/C* ^[^ ^40^ ^]^ and *CsgA/B/C/D* are significantly enriched and upregulated (Figure 3F; Figure S30, Supporting Information). The QS system, significantly enriched among upregulated genes, enables microorganisms to adapt to environmental conditions. In Gram‐negative bacteria, N‐acylhomoserine lactone (*HSL*) acts as a QS signaling molecule. Though *E. coli* lacks *HSL* synthesis, it detects *HSL* through the transcription factor *SdiA*.^[^ ^41^, ^42^, ^43^ ^]^ The increased expression of *SdiA* (Figure 3F) suggests its role in activating regulatory signals and promoting biofilm formation. Genes associated with the c‐di‐GMP pathway (*pdeA/L/K/H*) and bacterial adhesion (*sfmH*, *csgF*, *csgE*, *csgD*) show significant upregulation (Figure 3F). These changes correlate with enhanced biofilm formation^[^ ^44^, ^45^ ^]^ and bacterial adhesion, promoting *E. coli* growth and reproduction by increasing *c‐di‐GMP* expression and chemotaxis. In addition, OMVs also in the structural integrity^[^ ^46^, ^47^ ^]^ of bacteria and cell membrane biosynthesis^[^ ^48^ ^]^ related genes for the *E. coli* growth. The upregulation of ompA indicates enhanced membrane stability and integrity. Downregulated genes related to the cell periphery and component organization (Figure 3D) suggest OMVs influence bacterial components and biofilm formation, consistent with the modulation of QS and GAB‐ergic synapse.

Our hypothesis is that OMVs promote biofilm formation by upregulating genes mainly related to biofilm pathways. OMVs also stabilize bacterial membranes by upregulating *ompA*, while downregulation of genes related to cell periphery suggests OMVs influence biofilm formation and bacterial component regulation.

### 2.6 In Vitro Cytocompatibility Evaluation

**Figure 4:**
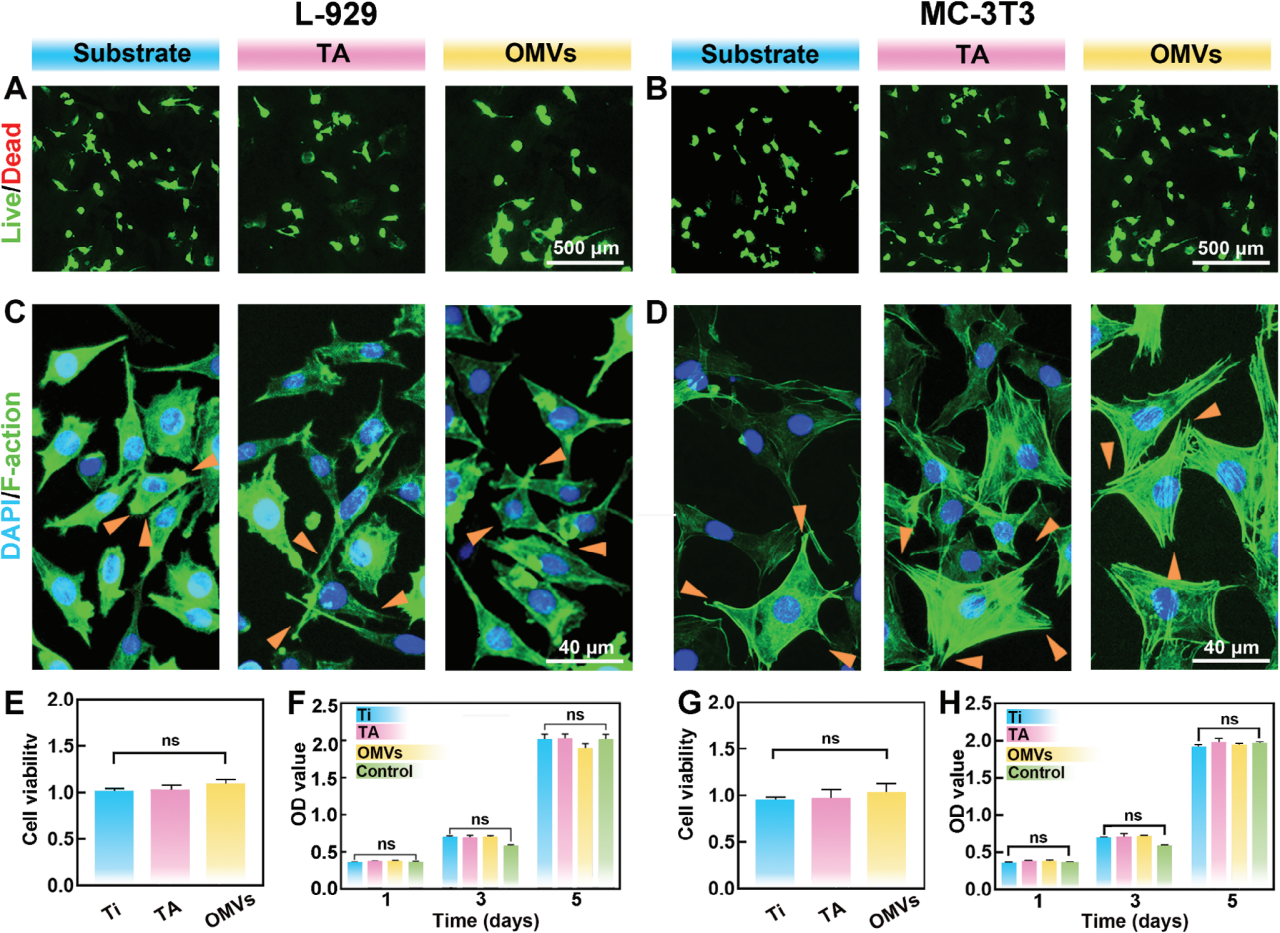
Cytocompatibility assessment of OMVs coating. A,B) LIVE/DEAD‐stained images of cell morphologies cultivated on Ti, Ti‐TA, and Ti‐TA‐OMVs on day 1. C,D) representative images of cell morphologies on day 1. Yellow arrows indicate the pseudopodia of cells. E,G) Cytotoxicity of L‐929 and MC‐3T3 by CCK8 on day 1. F,H) Cell proliferation of L‐929 and MC‐3T3 measured by CCK‐8. Data are presented as mean ± SD (*n* = 3) and analyzed using a one‐way ANOVA, **p < 0.01, ***p < 0.001, ns, no significance.

The mouse fibroblast cell lines (L‐929) and mouse embryonic osteoblast (MC3T3‐E1) cells were utilized to investigate the biocompatibility of various coatings in vitro. Substrates are recognized for their excellent biocompatibility, making them a prime choice for biomedical applications. LIVE/DEAD analysis reveals no dead cells in any of the groups, suggesting no significant toxicity in OMVs (**Figure** **4**A,B). Moreover, we observe the morphology and cytoskeletal structure of the treated cells after staining with FITC‐Phalloidin/DAPI. We discovered that cells in the OMVs group displayed clear nuclei with normal cytoskeletal structure similar to cells in the control group.

Overall, our findings suggest that our coatings have superior cytocompatibility and do not adversely affect cell proliferation or cell structure (Figure 4C,D). In addition, we evaluate cell toxicity and proliferation, along with the cell counting kit‐8 (CCK‐8) assay. Our findings reveal no significant difference in L929 cell viability between the Substrate, TA, OMVs, and control groups (Figure 4E). For MC3T3‐E1 cells, there are the same conclusions revealed in Figure 4G. On the contrary, the OMVs group demonstrates proliferation activity similar to that of the control group, suggesting that the coating of OMVs has no significant impact on cells (Figure 4F,H). The aforementioned findings demonstrate that OMVs, owing to their characteristics as phospholipid vesicles, possess excellent biocompatibility and can alleviate the adverse effects imposed by substrate and TA on cells. In addition, to further test the toxicity of the materials, LPS release content was tested. The results for the largest concentration of LPS under 0.1 ng mL^−1^ which is much lower than the inflammatory stimulus concentration 1 mg mL^−1^.^[^ ^49^, ^50^ ^]^ Therefore, the OMVs coating has good biocompatibility (Figure S39, Supporting Information).

### 2.7 In Vivo Assessment of Wound Healing Promotion

**Figure 5:**
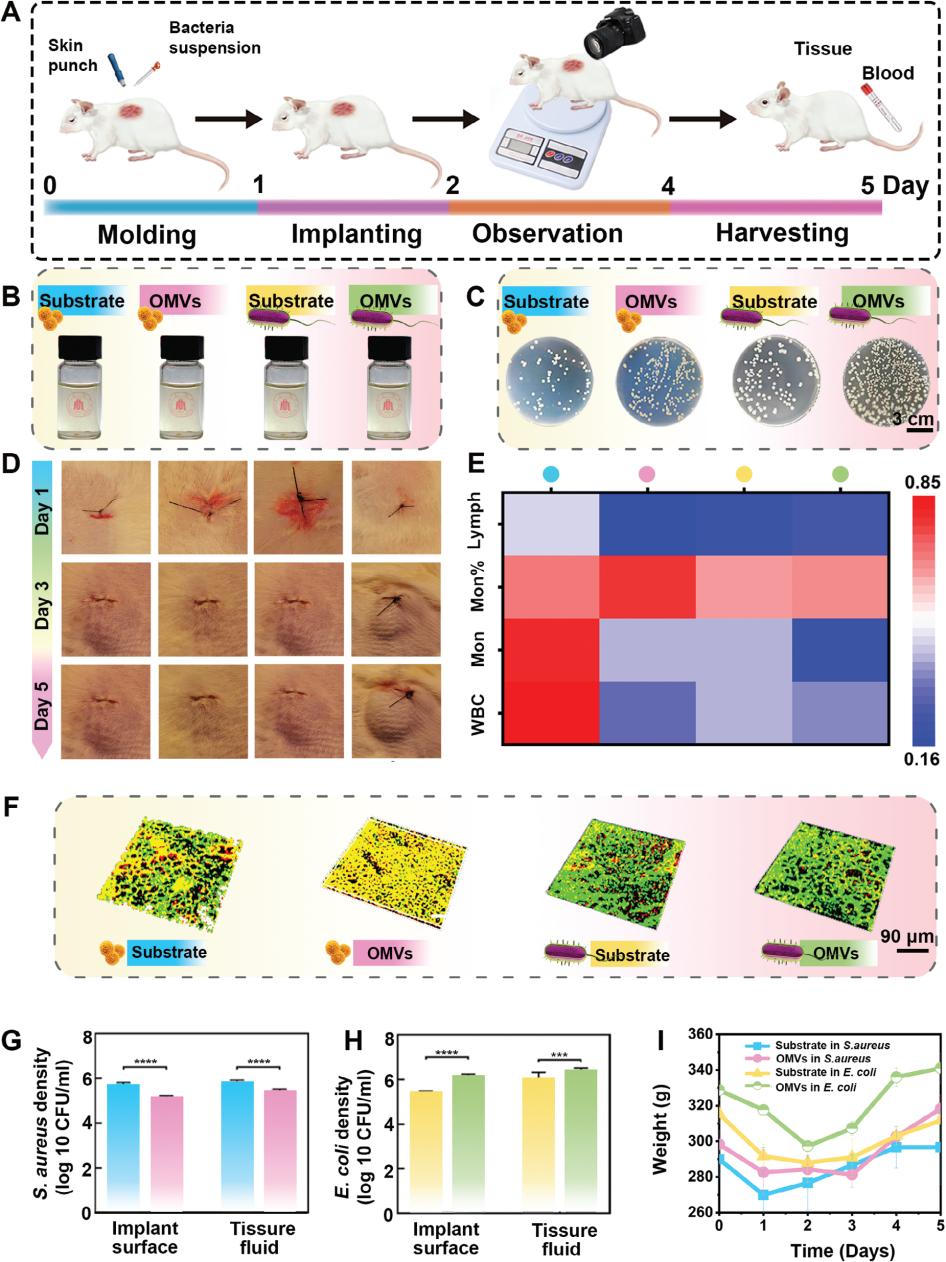
Evaluation of bacteriostatic and wound healing properties of OMVs coating in vivo. A) In vivo experimental procedure. B,C) Pictures of the *S. aureus* and *E. coli* colonies remaining in implant surface and exudate. D) Pictures of the healing process in infected wounds of rats. E) Heatmap of Serum Biochemical Indexes in Rats on the Day of Sacrifice. F) Quantitative red fluorescence analysis of implant surface. Pictures of the *S. aureus* G) and *E. coli* H) colonies remaining in implant surface and exudate. I) Changes in body weight in different groups of rats. Data are presented as mean ± SD (*n* = 3) and analyzed using a one‐way ANOVA, **p < 0.01, ***p < 0.001, ns, no significance.

The bacteriostatic efficacy and wound‐healing impact of the OMVs coating were examined through full‐thickness subcutaneous implant models infected with *S. aureus* and *E. coli* (**Figure** **5**A). Comparable outcomes were observed in mice through subcutaneous implantation of silicone sheets or titanium sheets. First, by collecting the exudate at the wound site and collecting the bacteria on the surface of the silicon substrate and OMVs, the spread plate method was used to evaluate the amount of the remaining bacteria (Figure 5B,C,G,H). In the OMVs groups, the number of remaining *S. aureus* in the exudate and implant surface is significantly reduced. On the contrary, the number of *E. coli* in the exudate and implant surfaces is significantly increased. The bacterial density in the exudate of OMVs in *S. aureus*, Substrate in *S. aureus*, OMVs in *E. coli*, and Substrate in *E. coli* are respectively 5.43 ± 0.02 lg CFU mL^−1^, 6.04 ± 0.11 lg CFU mL^−1^, 6.40 ± 0.47 lg CFU mL^−1^ and 6.17 ± 0.24 lg CFU mL^−1^, respectively. The bacterial density on the implant surface also reveals similar results. The same conclusion is reached regarding antimicrobial rates. As illustrated in Figure S31A (Supporting Information), the bacteriostatic efficacy of the titanium plate‐based group in the *S. aureus* infection model reveals that OMVs exhibit a higher bacteriostatic rate and exert a more robust regulatory effect on the subcutaneous flora of animals. However, contrasting results are observed in the *E. coli* subcutaneous infection model (Figure S31B, Supporting Information).

The inhibition of OMVs against *S. aureus* and the promotion of OMVs against *E. coli* is ulteriorly demonstrated. Subsequently, LIVE/DEAD Staining was employed to confirm the bacterial count remaining on the silicone sheets (Figure 5F). In rats infected with *S. aureus*, it is demonstrated that more bacteria were stained red in the OMVs group than in the Substrate group. In contrast, no more dead bacteria appear in the *E. coli* group than in the control group. Consistent findings are replicated following LIVE‐DEAD Staining of the implant surface utilizing titanium plates. The results reveal that in the Substrate‐*S. aureus* group, more viable bacteria persisted compared to the OMVs‐*S. aureus* group (Figure S32A, Supporting Information). Conversely, in the *E. coli* group, an inverse conclusion is reached (Figure S32B, Supporting Information). The relative quantitative analysis of red fluorescence is consistent with the previous results (Figure S33, Supporting Information). In addition, representative images of wounds were taken on days 1, 3, and 5. In the *S. aureus*‐infected model, the results indicate that compared with the Substrate group, the wounds in the OMVs group show less swelling and more complete healing of sutures (Figure 5D; Figure S34, Supporting Information). However, in the subcutaneous *E. coli*‐infected model, there is no significant difference in the healing degree between the Substrate and the OMVs, and even more obvious swelling appeared in the OMVs group. This suggests that OMVs have a good effect on the subcutaneous healing of *S. aureus* infections. In contrast, there is no therapeutic effect on the rat model of *E. coli* infection and it even aggravates the disease condition. The all above results are consistent with the exploration of the mechanism of selective bacteriostatic activity of OMVs.

### 2.8 Biotoxicity Assessment In Vivo

Major organs from Sprague‐Dawley (SD) rats with *S. aureus*‐infected wounds, treated with OMVs, were extracted and assessed for biotoxicity through hematoxylin and eosin (H&E) staining (Figure S35, Supporting Information). The results suggest that none of the groups show marked pathological abnormalities or damage. Furthermore, no obviously significant differences in body weight or the number of erythrocytes and neutrophils are observed between the Substrate and OMVs groups monitored on days 1, 2, 3, 4, and 5 (Figure 5E,I; Figure S36, Supporting Information). The above results show that the OMVs have good biocompatibility and would not cause organ damage and systemic inflammation.

### 2.9 In Vivo Evaluation of Histopathological Analysis

**Figure 6:**
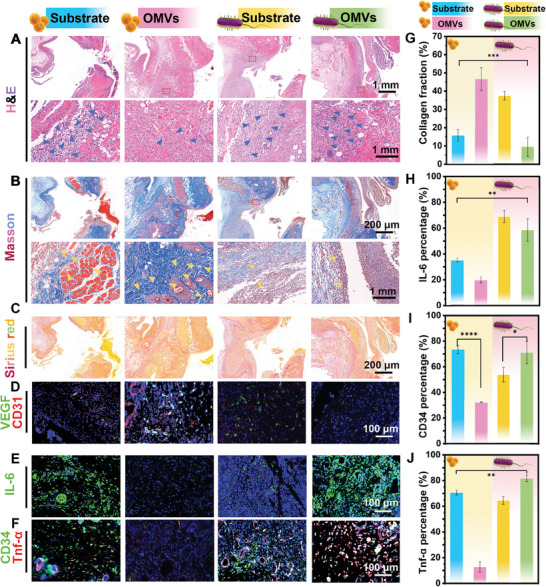
In vivo evaluation of histopathological analysis. A) H&E staining results of skin tissues after different treatments. B) Masson's trichrome staining results (yellow arrows represent the new vessels) and G) the corresponding quantitative collagen area fraction data. C) Sirius red staining images of the epidermal histological section. D) Representative immunohistochemistry images for VEGF and CD31. E) Representative immunohistochemistry images for IL‐6, F) Representative immunohistochemistry images for CD34 and TNF‐α. The relevant quantitative analysis of H) IL‐6, I) CD34 J) TNF‐α positive signals. Data are presented as mean ± SD (*n* = 3) and analyzed using a one‐way ANOVA, **p < 0.01, ***p < 0.001, ns, no significance.

The healing process of bacteria‐infected wounds involves several stages, including the bacteriostatic stage, reduced inflammation, angiogenesis, and collagen deposition. Observations were initially conducted on subcutaneous silicone‐implanted soft tissue models. We first evaluated the tissue inflammation response of the OMVs using H&E staining (**Figure** **6**A). The control substrate alone infected with *S. aureus* is denoted as Substrate‐*S. aureus* (denoted by the color blue), while the OMVs infected with *S. aureus* are labeled as OMVs‐*S. aureus* (denoted by the color pink); Similarly, the control implant model of the substrate alone infected with *E. coli* is referred to as Substrate ‐*E. coli* (denoted by the color yellow), and the OMVs infected with *E. coli* are designated as OMVs‐*E. coli*. It shows that a large number of inflammatory cells are clustered in Substrate‐*S. aureus*, Substrate ‐*E. coli*, OMVs‐*E. coli*, while the OMVs‐*S. aureus* group exhibiting minimal inflammatory cell infiltration and intact skin structure. These findings suggest an anti‐inflammatory role of OMVs in the OMVs‐*S. aureus* group wound infection model. Apart from inflammation, collagen deposition and angiogenesis also play a critical role in wound healing, which we evaluated using Masson's trichrome and Sirius red staining (Figure 6B,C). As shown in Figure 6B, more abundant new vessels and collagen are observed in the OMVs‐*S. aureus* group, which represents better wound healing than the other groups. The result of Sirius red staining also shows that there are more mature type I collagen fibers in the OMVs‐*S. aureus* group, but less mature fibers in the OMVs‐*E. coli* group and the substrate group in the *E. coli*‐infected skin wounds (Figure 6C). In addition, a subcutaneous implant model of titanium substrates was also applied. HE, Masson, and Sirius red staining all yielded the same results as described above (Figure S37A–C, Supporting Information). This confirms that the OMVs coating has the same effect in the case of different substrates.

The Immunohistochemistry (IHC) assay was used to assess the inflammation response. Vascular endothelial growth factor (VEGF) and CD31 play a pivotal role in fostering the proliferation, migration, and angiogenesis of vascular endothelial cells. They are frequently employed as an indicator to assess angiogenesis in the context of wound healing. In the OMVs‐ the *S. aureus* group, exhibits the highest intensity of both red and green fluorescence (Figure 6D). Additionally, utilizing IL‐6, Cluster of Differentiation 34 (CD34 and TNF‐α as inflammatory markers in two disease models. In the *S. aureus* infective model, the positive expression of Interleukin‐6 (IL‐6) increased in the control group more than in the OMVs group (Figure 6E). CD34 and TNF‐α, recognized as typical cellular inflammatory factors, demonstrate abundant expression during an inflammatory response. As depicted in the Figure 6F, the OMVs‐*S. aureus* group exhibits lower expression levels of CD34 and TNF‐α, whereas the other three groups display a significant and plentiful expression of these inflammatory markers. The relief of the inflammatory response depends on the oxidative stress and metabolic disturbances caused by the OMVs to *S. aureus*. Quantitative statistics also reveal similar results (Figure 6G–J). Additionally, samples on titanium substrates were labeled with TNF‐α and CD34. The results obtained with titanium substrates parallel those obtained with silica gel substrates. This concurrence is further supported by the corresponding quantitative statistics presented in Figure S37C–I (Supporting Information). Relevant quantitative statistics are summarized as radar plots illustrating significant differences between groups (Figure S38, Supporting Information). These results illustrate the significant benefits of OMVs across various stages of wound healing, including inflammatory reactions, cell proliferation, and soft tissue repair in the *S. aureus* model, but the OMVs‐*E. coli* group shows the opposite effect, delaying wound healing. Ultimately, we demonstrate that the coating of OMVs has completely opposite effects in two types of animal models with different bacterial infections. The results confirm the aforementioned bacteriostatic test results, mainly due to the OMVs^’^ selective effect on the bacterial population response as well as the QS system.

## 3 Conclusion

Inspired by the communication of information transmitted between bacteria, OMVs derived from *E. coli* are used as a highly selective bacteriostatic substance to establish a stable implant coating with the assistance of a polyphenol layer (poly (tannic acid)) to regulate the microecological balance under bacterial infection conditions. In vitro experiments demonstrate that the OMVs coating inhibits the growth of heterogeneous bacteria while promoting the growth of homogeneous bacteria. In vivo, experiments further reveal that the OMVs coating yields distinct therapeutic outcomes, capable of healing wounds infected with heterologous bacteria but exacerbating infections in wounds with homologous bacteria. Through high‐throughput sequencing and bioinformatics analysis, we have clarified that the selective bacteriostatic capability of the coating arises from the bioactive components encapsulated in the OMVs. Primarily, these components regulate bacterial metabolism by up‐regulating anti‐oxidative stress genes in heterologous bacteria and activating biomembrane‐related genes in the parent bacteria. This bioinspired natural “angel and devil” OMVs coating presents a highly selective bacteriostatic intervention through biological pathways, holding significant potential in nurturing a healthy microbial ecosystem.

## 4 Experimental Section

### Materials

Commercial Ti implants, consisting of two types of Ti disks (8 × 8 × 0.2) mm^3^ and (Φ0.8 mm × 0.2 mm), were procured from Shengze Metal Ltd (Baoji, China). Commercial silicone sheets: consisting of two types of silicone disks (8 × 8 × 0.2) mm^3^ and (Φ0.8 mm × 0.2 mm), were procured from Jinhua, China. Commercial Hap sheets consisting of two types of hydroxyapatite disks (8 × 8 × 0.2) mm^3^ and (Φ0.8 mm × 0.2 mm), were procured from Bayamon Bioactive Materials Co., LTD. TA was purchased from Aladdin (USA). Glutaraldehyde was obtained from J&K Scientific (USA). Deionized water was obtained from pure water systems (Barnstead GenPure xCAD Plus, Thermo Fisher Scientific, USA). All chemical reagents were purchased by Baoxin Biotechnology Ltd (Baoke, China) unless stated otherwise.

### OMVs Isolation

OMVs were isolated using a combination of ultrafiltration and the ultracentrifugation method. Initially, a centrifuge (5100 g, 4 °C, 20 min) was used to remove bacterial cells and debris. The solution was then filtered using 0.22 µm sterile filters and the resulting liquid was centrifuged in a 100 kDa ultrafiltration tube (4 °C, 3000 g, 20 min) to obtain purified OMVs solution. Next, the solution was subjected to ultracentrifugation at 100 000 g for 2 h at 4 °C, and the sediment collected from underneath was collected. Finally, the pure OMVs were suspended in 0.9% NaCl solution and stored at −20 °C.

### Bacterial Cultural

To fabricate OMVs, gram‐negative *E. coli* (ATCC 25 922) was applied. Bacteria were cultured in Luria‐Bertani (LB) broth and Brain Heart Infusion Medium (BHI). The concentration of samples was determined using the Optical Density at 660 nm (OD_660_ = 0.53 is equivalent to 1 × 10^9^ CFU mL^−1^).

### OMVs Characterization

To prepare for TEM analysis, OMVs were harvested from *E. coli* and placed on 400‐mesh copper grids. The grids were then stained using 2% uranyl acetate and imaged using a JEM1011 microscope (JEOL, Japan), with an accelerating voltage of 100 kV. In addition, dynamic light scattering and zeta potential analysis were conducted on the OMVs with a Zetasizer 3000HSA instrument (Malvern Instruments Limited, Britain), which measured their size and zeta potential. Finally, the overall protein content of the OMVs was evaluated using a BCA protein assay kit on day1,4 and 7 (Beyotime, China).

### Fabrication of Substrate‐TA‐OMVs

To prepare the substrate for use, they were immersed in a sequence of cleaning solutions consisting of acetone, ethanol, and distilled water for 20 min, followed by air drying. The fabrication process consisted of immersing the cleaned substrates in a TA solution (2 mg mL^−1^) and irradiating them under a UV lamp (280 nm, 6 mW cm^−1^) for 4 h. The resulting Substrate‐TA were then rinsed with 200 µL of deionized water, air‐dried, and immersed in OMVs solution extracted from *E. coli* suspensions for 72 h at room temperature to obtain the Substrate‐TA‐OMVs.

### Surface Characterization

The surface chemical structure of substrates was assessed to confirm the successful coating of TA and OMVs on the substrates. Experiments were conducted on titanium, silicone, and hydroxyapatite substrates. The spectra within the range of 500 to 4000 cm^−1^ with a resolution of 4 cm^−1^ were acquired with ATR‐FTIR (Nicolet 6700, Thermo Scientific TM, USA) after substrate drying. The surface element variation of each sample was analyzed through XPS (Thermo Scientific K‐Alpha, USA) within the range of 100–1349 eV using 150 eV pass energy. The morphology of each sample was also assessed through SEM (Inspect F50, FEI, USA). The surface of roughness was detected via AFM (Dimension, Bruker, Germany). Furthermore, the water contact angle of each implant was assessed using the sessile drop method (Drop Shape Analyser‐DSA25, Kruss, Germany). 1 µL of water was used as the working medium, and the images were collected after the water droplets were placed on each sample for 0.5 s. The measurements were repeated three times at different locations.

### Stability of OMVs Coating

To preserve its biological activity, OMVs were stored at −20 °C without repeated freeze‐thaw cycles and used for stability testing. The coating materials were either used immediately after production or briefly stored at 4 °C before use. Through these storage methods, the stability of the coating was effectively assessed. For the samples with OMV, DiD‐stained OMVs were used to analyze morphological stability. The acquired samples were examined using an inverted fluorescence microscope (IX71S1F‐3, OLYMPUS, Japan) on days 1, 7, 14, and 21, with each sample being redyed at every tested time point. The spectra within the range of 500 to 4000 cm^−1^ with a resolution of 4 cm^−1^ were acquired with ATR‐FTIR (Nicolet 6700, Thermo Scientific TM, USA) after substrate drying. The surface element variation of each sample was analyzed through XPS (Thermo Scientific K‐Alpha, USA) within the range of 100–1349 eV using 150 eV pass energy. XPS and FTIR were employed to assess the stability of the OMVs coating on days 1, 4, and 7, respectively. Additionally, the plate counting method assesses the impact of implants on bacterial activity by quantifying the colony‐forming units (CFU) at different time points. The OMVs were coated and stored for varying durations. Stability was assessed by counting colonies after consistent incubation and growth periods (37 °C, 24 h). The antibacterial properties of OMVs were evaluated against *S. aureus* and *E. coli* on days 1, 4, and 7, respectively.

### Bacteria Culture

The Gram‐positive bacterium *S. aureus* (ATCC 29 213), *S. epidermidis* (ATCC 12 228), as well as the Gram‐negative *E. coli*, and *P. aeruginosa* (ATCC 27 853) were cultured in LB agar plates, while *E. faecalis* (ATCC25922) was cultured on BHI agar plates.

### Assessment of Bacterial Viability

Silicone sheets and titanium (Ti) were employed as two substrates for in vitro antibacterial experiments. The plate counting method was used to evaluate the effects of different implants on bacterial activity by determining the number of CFUs. Samples (Substrate, Substrate‐TA, Substrate‐TA‐OMVs) were placed in a 24‐well plate, and a bacterial suspension of *S. aureus*, *E. coli*, *E. faecalis*, *S. epidermidis* or *P. aeruginosa* (1 mL, 1 × 10^6^ CFU mL^−1^) was added, respectively. The pure OMVs suspension (OMVs (−)) was co‐cultured with *S. aureus*, *E. coli*, *S. epidermidis*, *P. aeruginosa*, or *E. faecalis*, to assess the relative impact of Substrate‐TA‐OMVs (OMVs (+)) compared to pure OMVs (OMVs (−)) in in vitro bacterial experiments. The plate was then incubated at 37 °C for 24 h, followed by discarding the medium and rinsing the samples with phosphonate buffer solution (PBS). The implants were then immersed in 2 mL of PBS and ultrasonically detached for 20 min. The resulting bacteria suspension was diluted 10^4^‐fold and distributed evenly onto agar plates with 50 µL, which were then incubated at 37 °C for 24 h. Representative images of the culture plates were captured using a digital camera and the quantity of bacteria was measured. The relative bacterial ratio of the samples was computed using the following formula: the number of CFUs of each group was counted.

(1) $$Log\;reduction=\mathrm{lg}\left(CFU/50\;\mu L\right)$$

(2) $$\mathrm{Bacterial}\mathrm{survival}\mathrm{ratio}\left(\%\right)=\mathrm{CFU}\mathrm{of}\mathrm{experimental}\mathrm{groups}/\mathrm{CFU}\mathrm{of}\mathrm{control}\times 100\%$$

### Bacterial Morphology Observation

The antibacterial properties of silicone sheets and Ti‐based substrates were tested using the LIVE/DEAD BacLight bacteria viability kit (Thermo Fisher Scientific, USA). Each group of samples was incubated in 1 mL of bacterial suspension containing 1 × 10^6^ CFU mL^−1^ at 37 °C for 24 h. After incubation, the samples were washed with PBS to remove non‐adherent or loosely adherent bacteria. The samples were then stained using the LIVE/DEAD BacLight bacteria viability kit as per the instrument specifications and examined using a Confocal laser scanning microscope (CLSM, Leica TCS SP5II, Germany). In order to further observe bacterial attachment on the surfaces, the Ti‐based Substrates co‐incubated with bacterial suspension were fixed with 2.5% glutaraldehyde (Solarbio, China) overnight at 4 °C and dehydrated using graded concentrations of alcohol (ranging from 30% to 100% v/v). The sample surfaces were sputtered and coated with Au, and SEM (Inspect F50, FEI, USA) was used to observe the samples at magnifications of 5000 to 20 000 times, under an accelerated voltage of 3 kV.

### In vitro Study on the Selective Antibacterial Mechanism of OMVs

To further investigate the precise selective antibacterial mechanism of OMVs. The OMV obtained from *E. coli* was first freeze‐thawed repeatedly, then ground 30–40 times with a glass homogenizer, and then shaken with ultrasonic. To ensure complete disruption of the OMVs, their morphology was examined by TEM after ultrasonic shock, and their particle size and zeta potential were assessed with DLS. Then they were extruded using a mini extruder (LF‐1, Avestin, Canada) employing a 200 nm polycarbonate porous membrane to obtain a spherical structure.^[^ ^51^, ^52^ ^]^ Then, using the same method as before, the OMVs were deposited on the surface of the substrate to obtain Substrate‐TA‐DOMVs. DiD‐staining was used to test DOMVs covered by success on the substrate. SEM was used to characterize the morphology of bacteria, and plate counting was used to detect the survival rate of bacteria.^[^ ^53^, ^54^ ^]^

### Photothermal Properties Evaluation

A high‐precision thermal infrared imager was employed to capture the thermographic profiles of different samples, followed by an intricate analysis of these thermographic data using the HIKMICRO Analyzer. Substrate, Substrate‐TA ‐TA, and Substrate ‐TA‐OMVs were exposed to NIR (808 nm, 1.0 W cm^−2^) to elucidate the impact of OMVs. Throughout the temperature escalation phase, temporal temperature variations were meticulously documented at 30 intervals over a duration of 10 min. Concurrently, during the temperature descent phase, recordings were made every 5 s for 2 min. Moreover, the photothermal stability of different samples was tested over three heating‐cooling cycles of 10 min each. Finally, the thermal diffusivity of different samples was detected by the Flash Method Laser Thermal Conductivity Analyzer (LFA 467 HyperFlash, NETZSCH, Germany).

### Cell Culture

L‐929 and MC3T3‐E1 cell lines were procured from the American Type Culture Collection (ATCC). Both cell lines were cultured in Dulbecco's Modified Eagle's Medium (α‐MEM), supplemented with 10% fetal bovine serum (FBS) and 1% penicillin/streptomycin, at 37 °C with 5% CO_2_. The initial cell density of all samples was 1 × 10^4^ cells/mL unless otherwise specified.

### Cell Proliferation Activity

Different titanium‐based substrates were added to the complete culture medium in 24‐well plates, and after incubating for 24 h, different extracts were obtained. The toxicity and proliferation activity of L‐929 and MC3T3‐E1 cells were assessed by means of the CCK‐8 (APE×BIO, USA). Notably, the cell proliferation activity assay used an initial cell density of 0.5 × 10^4^ cell mL^−1^. A total of 1 mL of cell suspension was added to each of the 24‐well plates, and the cells were then cultured for 24 h. After this, cells were treated with different extracts, and the proliferative activity of cells and cytotoxicity were evaluated using the CCK‐8 reagent at predetermined time points. The cell culture medium containing 10% CCK‐8 reagent and cells were co‐incubated in the dark for 4 h. For the CCK‐8 test, cells were measured at 450 nm using a microplate reader (Multiskan SkyHigh, Thermo Fisher, America). Cells cultured on the pure titanium disks were considered as the control. The cell viability (%) of cells was calculated using the following formula:

(3) $$\mathrm{Cell}\;\mathrm{viability}=100\%\times \left(OD_{\mathrm{test}}-OD_{\mathrm{blank}}\right)/\left(OD_{\mathrm{control}}-OD_{\mathrm{blank}}\right)$$

where OD_test_, OD_blank_, and OD_control_ are the values of the well with coatings (Ti‐TA‐OMVs, pure medium, and Ti groups) respectively.

### Observation of Cell Morphology and Cytoskeleton Structures

The morphology of various cells treated with different extracts was evaluated. 1 mL of cell suspension (0.5 × 10^4^ cells mL^−1^) was added to 24 well plates and treated with various extracts for 24 h. Subsequently, the samples were rinsed twice with PBS and fixed with a 4% formaldehyde tissue fixation solution for 1 h. Then, Triton X‐100 (0.1% v/v) was applied to penetrate the internal cells for 20 min, and FITC‐phalloidin (Solarbio) and 4′,6‐diamidino‐2‐phenylindole (DAPI, Solarbio, China) were utilized to counterstain cytoskeleton and nucleus of cells. Finally, the fluorescent images were captured using a fluorescence microscope (DM8, Leica, Germany). The toxicity of the material was further determined. The fabricated Substrate‐TA‐OMVs were immersed in 10 mL PBS buffer (pH 7.2). Then slowly stirred at 37 °C. At the same point in time on days 1, 4, and 7, 1 mL samples were withdrawn from the solution. Immediately after each sampling, the same volume of fresh‐release medium was replenishable to keep the total fluid volume constant. Briefly, the obtained liquid containing OMVs was added to an LPS antibody‐coated 96‐well plate. Following the manufacturer's instructions, the solutions were mixed, incubated, washed, and then a color reagent was added. The UV–vis absorbance was measured at 450 nm using a microplate reader (Synergy H1, BioTek, Winooski, VT, USA).

### LIVE/DEAD Staining

The LIVE/DEAD staining was utilized to evaluate the toxicity of various samples. A 1 mL cell suspension was added to 24‐well plates and treated with various extracts for a duration of 24 h. The samples were stained according to the instructions of the Calcein‐AM/PI double staining kit (Solarbio) and typical images were recorded using a fluorescence microscope (DM8, Leica, German).

### Construction of Subcutaneous Infection Model and Samples Implantation

All animal experiments in this study were approved by the Animal Care and Experiment Committee of the West China Hospital of Sichuan University (WCHSIRB‐D‐2022‐186) and conducted in accordance with the guidelines outlined in the National Research Council's Guide for the Care and Use of Laboratory Animals. Animal experiments were approved by the Institutional Animal Care and Use Committee (IACUC) of Sichuan University. Briefly, the male SD rats (weight: 180–220 g) were divided randomly into 4 groups (*n* = 10). Anesthesia was administered by using 2% isoflurane (0.3 mL kg^−1^), following which the back of the rats was shaved and dorsal wall skin was exposed. Two incisions, each with a diameter of 0.8 cm, were made along the sides of the spine to establish a subcutaneous pocket model. The titanium and silicone implant models were implanted into the animals respectively. Subsequently, a bacterial suspension (20 µL, *S. aureus*, 1 × 10^8^ CFU mL^−1^) or (20 µL, *E. coli*, 1 × 10^8^ CFU mL^−1^) was injected into each pocket wound. Two wounds on the back of each rat were injected with the same bacterial solution. Substrate and Substrate‐TA‐OMVs were implanted into the subcutaneous tissue of rats. The rats infected with *S. aureus* after implantation of substrate were designated as Substrate ‐*S. aureus*, while the injection of Substrate‐TA‐OMVs was named OMVs‐*S. aureus*. The rats infected with *E. coli* after implantation of substrate were appointed as Substrate ‐*E. coli*, and the injection of Substrat‐TA‐OMVs was designated OMVs‐*E. coli*. After the procedure, the subcutaneous tissue and skin were sutured. The rats were then cultured for 1, 3, and 5 days, respectively.

### In vivo Bacterial Survival Tests

To evaluate the bacterial survival rate of different samples after implantation, plate counting was utilized to calculate the number of colonies after 24 h. On day 1, three rats in each group were euthanized, and sterile swabs were used to collect the exudate around the wound. Subsequently, the swabs were placed into a centrifuge tube containing sterile LB. At the same time, the round Ti disks were removed from the wound and immersed in one milliliter of sterile LB. Following equalization, the evenly diluted wound exudate was inoculated onto LB agar plates to examine the remaining bacteria. Additionally, LB medium containing Substrate and Substrate‐TA‐OMVs was subjected to ultrasonic vibration, followed by a 100‐fold dilution of the medium, and then inoculated onto LB agar plates. To further evaluate antibacterial activity, three samples from each group were selected for LIVE/DEAD staining, following the same steps as the in vitro experiments. The typical images of the wound status were also recorded with a digital camera on days 1, 3, and 5. Blood samples were taken to investigate neutrophil and hemoglobin levels on the corresponding days.

### Histopathological Analysis

On the 5th day after treatment, the rats were anesthetized and euthanized. Samples from the wound sites were then excised and fixed in a 4% paraformaldehyde solution. Samples for neutrophils and hemoglobin measurements were obtained by collecting 5 mL of blood from the abdominal aortic into a polypropylene tube containing plasma heparin. Skin samples were embedded in paraffin and sectioned into 6 µm thick slices. Subsequently, H&E, Masson's trichrome staining and Sirius red staining were conducted. Additionally, two inflammatory factors, CD34 and TNF‐α, were labeled using IHC staining. Finally, a comprehensive assessment of toxicity was carried out on vital organs, encompassing the heart, liver, spleen, lungs, and kidneys.

### RNA Sequencing

The effects of OMVs on bacteria were analyzed using RNA‐Seq. Specifically, bacteria treated were compared with OMVs to untreated bacteria, focusing on *S. aureus* and *E. coli*. Equal amounts (500 µL) of OMVs and PBS were respectively added to bacteria growing in liquid culture in a healthy state and cultured at 37 °C for 24 h, and then detected by RNA‐seq. The three parallel samples were prepared for each group and Illumina sequencing using the TruSeqRNA sample preparation Kit from Illumina and NovaSeq 6000 sequencing (150bp × 2, BIOZERON, China). The Genome Database was used to analyze the RNA sequencing (https://www.ncbi.nlm.nih.gov/genome/?term=ASM1342v1 or https://www.ncbi.nlm.nih.gov/genome/?term=ASM584v2). The edgeR software was used to identify differentially regulated genes and statistical significance was defined as *p* < 0.05. Genes that exhibited at least a two‐fold differential regulation compared to the control were exclusively taken into consideration.

### Statistical Analysis

The experimental data is presented as the mean value with standard deviation (SD). Each experimental group comprises a minimum of 3 replicates (*n* ≥ 3). Statistical analysis of the data was conducted using Origin 2021 software, utilizing one‐way analysis of variance (ANOVA) and two‐way ANOVA methods. The significance levels for statistical analysis were defined as follows: *p < 0.05, **p < 0.01, ***p < 0.001, ****p < 0.0001, and ns *p* > 0.05, respectively.

## Conflict of Interest

The authors declare no conflict of interest.

## Supporting information

Supporting Information

## Data Availability

The data that support the findings of this study are available from the corresponding author upon reasonable request.
