# Supplementary material for: Exploring Naturally Tailored Bacterial Outer Membrane Vesicles for Selective Bacteriostatic Implant Coatings
Source: Adv Sci (Weinh). 2024 Aug 21;11(39):2405764. doi: 10.1002/advs.202405764 (PMC11497020; doi:10.1002/advs.202405764)
Supplement: Supplementary file 1 — Supporting Information [file ADVS-11-2405764-s001.docx]

Supporting information

Exploring Naturally Tailored Bacterial Outer Membrane Vesicles for Selective Bacteriostatic Implant Coatings

Zilin Zhou,^1&^ Lizhong Sun,^1,2&^ Yuanyuan Tu,^1^ Yingming Yang,^1,3^ Ailin Hou,^1,3^ Jiyao Li,^1,3^ Jun Luo,^4^ Lei Cheng,^1,3^ Jianshu Li,^1,4,5^ Kunneng Liang,^1,3^* Jiaojiao Yang^1,6^*


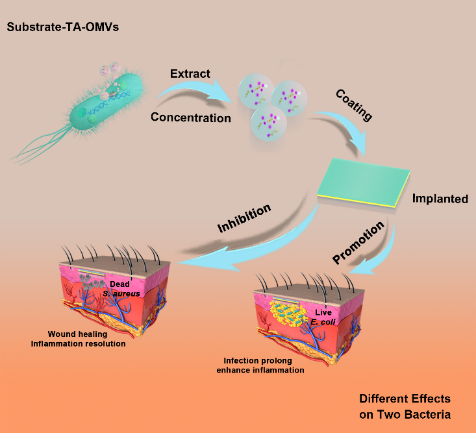


OMVs were first used to construct implant coatings, revealing an unexpected selective bacteriostasis ability at the macroscopic level. Employing a facile polyphenol bridging technology facilitated the stabilization of bacterial OMVs on diverse implant surfaces. This OMVs coating endowed implants with the ability for selective bacterial inhibition. This approach offers natural alternatives for potential selective antimicrobial interventions in infectious disease treatment.

**Captions of Figures**

**Figure S1**. Fabrication of Substrate-TA-OMVs and selective bacteriostatic studies in vivo. (A) Fabrication of Substrate-TA-OMVs and selective bacteriostatic studies in vivo. (Coordination bonds is drawn in the figure in the case of Ti and hydrogen bonding in the case of Si). (B) The in vivo selective bacteriostatic studies of Substrate-TA-OMVs using two different bacterial skin wound models, and elucidation of the underlying selective bacteriostatic mechanism.

**Figure S2**. SEM images of silicone, silicone -TA an silicone -TA-OMVs. (scale bar:5 μm)

**Figure S3**. AFM images of Substrate (Ti), TA and OMVs.

**Figure S4**. (A) X-ray Photoelectron Spectroscopy (XPS) spectra of Substrate (Ti), TA and OMVs. Core level spectra of (B) P 1s of OMVs, (C) C 1s of TA and (D) C 1s of OMVs.

**Figure S5.** Fourier transform infrared spectrometer (FTIR) spectra of Substrate (Ti), TA and OMVs.

**Figure S6**. (A) Bacterial colonies treated with Substrate, TA and OMVs. (scale bar: 2 cm). (B) Bacterial density of *S. aureus*, *E. coli* and *P. aeruginosa* treated with Substrate (Ti), TA and OMVs. The data are shown as the mean ± standard deviation (n = 3).

**Figure S7**. Bacterial density of *S. aureus, E. coli* and *P. aeruginosa* treated with different samples (Substrate (Ti), TA, OMVs).

**Figure S7**. Bacterial density of *S. aureus, E. coli* and *P. aeruginosa* treated with different samples (Substrate (Ti), TA, OMVs). Data are presented as mean ± SD (n = 3) and analyzed using a one-way ANOVA, ***p* < 0.01, ****p* < 0.001, ns, no significance.

**Figure S8**. (A) Bacterial colonies treated with Substrate (Ti), TA, OMVs. (B) Bacterial density of *S. epidermidis* and *E. faecalis* treated with different samples, the data are shown as the mean ± standard deviation (n = 3).

**Figure S9**. SEM images of *S. epidermidis* and *E. faecalis* cultured with Substrate (Ti), TA, OMVs

**Figure S10**. LIVE/DEAD-stained confocal images of *S. epidermidis* and *E. faecalis* cultured with Substrate (Ti), TA, OMVs.

**Figure S11**.LIVE/DEAD-stained confocal images of *S. aureus*, *E. coli* and *P. aeruginosa* treated with Substrate (Ti), TA, OMVs.

**Figure S12.** Quantitative analysis of fluorescence intensity of Substrate (Ti), TA, OMVs.

**Figure S13. Black frame**：(A) Images of bacterial colonies treated with each group in vitro. (B) SEM images of *S. aureus*. (C) and (D) Bacteriostatic ratio of *S. aureus* from spread plate results in vitro.

**Red frame：**(A) Images of bacterial colonies treated with each group in vivo. (B) SEM images of *S. aureus.* (C) and (D) Bacteriostatic ratio of *S. aureus* from spread plate results in vivo.

**Figure S14.Black frame**：Images of bacterial colonies treated with each group in vitro. (B) SEM images of *E. coli*. (C) and (D) Bacteriostatic ratio of *E. coli* from spread plate results in vitro.

**Red frame：**(A) Images of bacterial colonies treated with each group in vivo. (B) SEM images of *E. coli.* (C) and (D) Bacteriostatic ratio of *E. coli* from spread plate results in vivo.

**Figure S15.** (A) Images of bacterial colonies treated with each group in vitro. (B) SEM images of *P. aeruginosa*. (C) and (D) Bacteriostatic ratio of *P. aeruginosa* from spread plate results in vitro.

**Figure S16.** (A) Images of bacterial colonies treated with each group in vitro. (B) SEM images of *S. epidermidis*. (C) and (D) Bacteriostatic ratio of *S. epidermidis* from spread plate results in vitro.

**Figure S17.** (A)Images of bacterial colonies treated with each group in vitro. (B) SEM images of *E. faecalis*. (C) and (D) Bacteriostatic ratio of *E. faecalis* from spread plate results in vitro.

**Figure S18.** Photothermal effect evaluation.

**Figure S19.** Stability of the OMVs groups.

**Figure S20**. Characterization of DOMVs and DOMVs coating. (A) TEM images, and (B) diameter of Destroyed OMVs. (C) Zeta potential (D) DiD-stained of Substrate, TA and OMVs.

**Figure S21**. SEM images of *E. faecalis* and *S. epidermidis* cultured with Substrate, TA, DOMVs.

**Figure S22.** Bacterial density of *S. aureus, E. coli* and *P. aeruginosa* treated with Substrate, TA and DOMVs.

**Figure S23.** Ribosome contamination assessment of *S. aureus* exposed to OMVs.

**Figure S24.** Sample correlation analyses of *S. aureus* exposed to OMVs.

**Figure S25.** Volcano map for the distribution of DEGs

**Figure S26.** Heat map of DEGs **o**f *E. coli exposed* to *S. aureus***.**

**Figure S27.** Ribosome contamination assessment of *E. coli* exposed to OMVs.

**Figure S28.** Sample correlation analyses of *E. coli* exposed to OMVs.

**Figure S29.** Heat map of DEGs of *E. coli exposed* to OMVs.

**Figure S30.** Downregulated DEGs enriched in the KEGG pathway

**Figure S31.** (A) Bacterial density of *S. aureus* remaining in Ti based implant surface and exudate. (B) Bacterial density of *E. coli* remaining in Ti based implant surface and exudate. The data are shown as the mean ± standard deviation (n = 3).

**Figure S32.** LIVE/DEAD stained confocal images of *S. aureus* (A) and *E. coli* (B) on the surface of titanium Substrate and OMVs retrieved from rats.

**Figure S33.** Quantitative red fluorescence analysis of titanium implant surface.

**Figure S34.** Pictures of the healing process in infected wounds of titanium implant models.

**Figure S35**. Biotoxicity analysis: H&E staining results of major organs in *S. aureus* infected rats after different treatments.

**Figure S36**. Biotoxicity analysis: changes of body weight in different groups of titanium implant models.

**Figure S37**. Histopathologic analysis of slices of the titanium substrate mode.

**Figure S38.** Comprehensive performance of Substrate, TA, OMVs.

**Figure S39.** LPS content release on days 1, 4 and 7. (n=3)


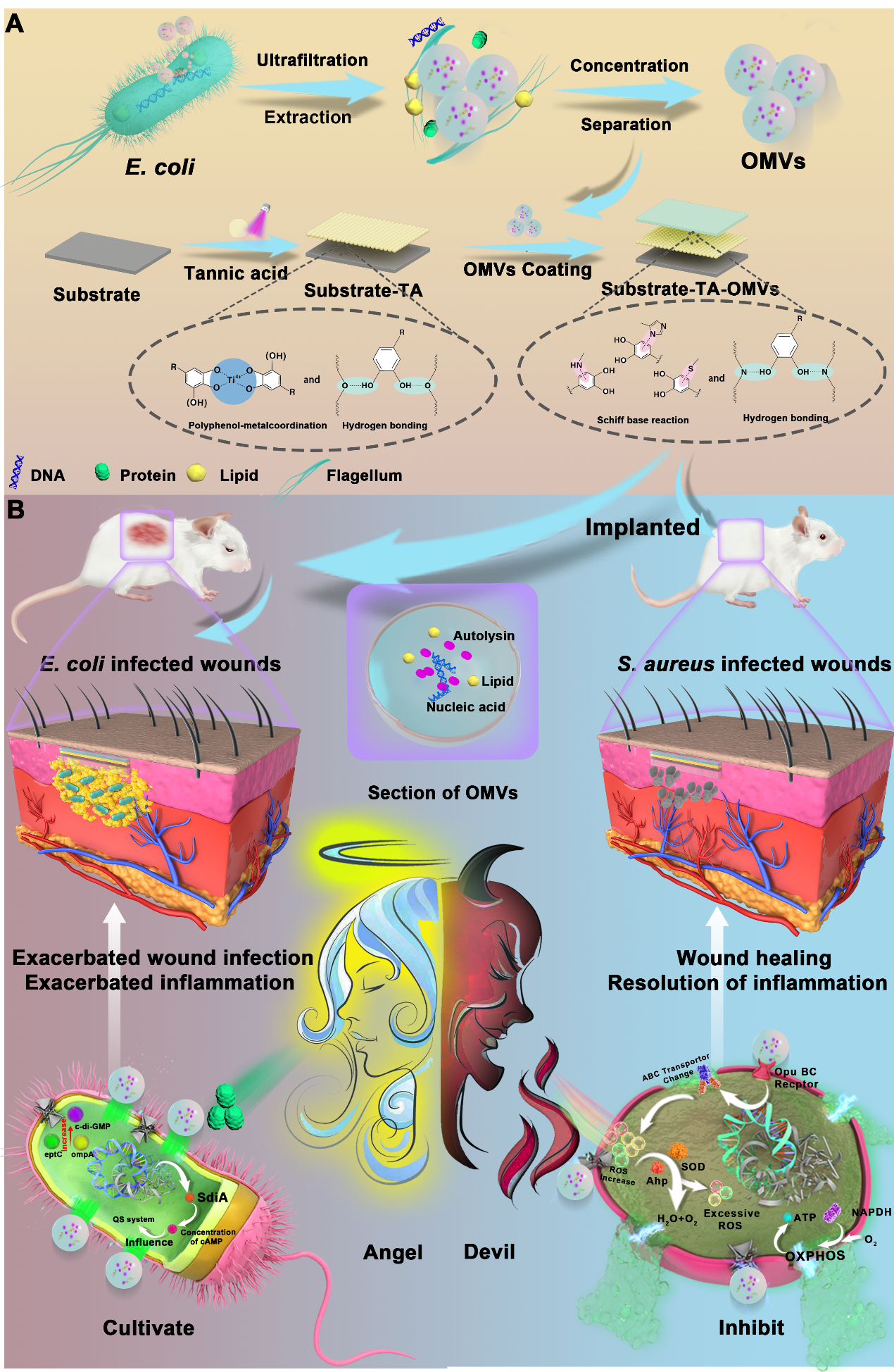


**Figure S1**. Fabrication of Substrate-TA-OMVs and selective bacteriostatic studies in vivo. (A) Fabrication of Substrate-TA-OMVs and selective bacteriostatic studies in vivo. (Coordination bonds is drawn in the figure in the case of Ti and hydrogen bonding in the case of Si). (B) The in vivo selective bacteriostatic studies of Substrate-TA-OMVs using two different bacterial skin wound models, and elucidation of the underlying selective bacteriostatic mechanism.

**
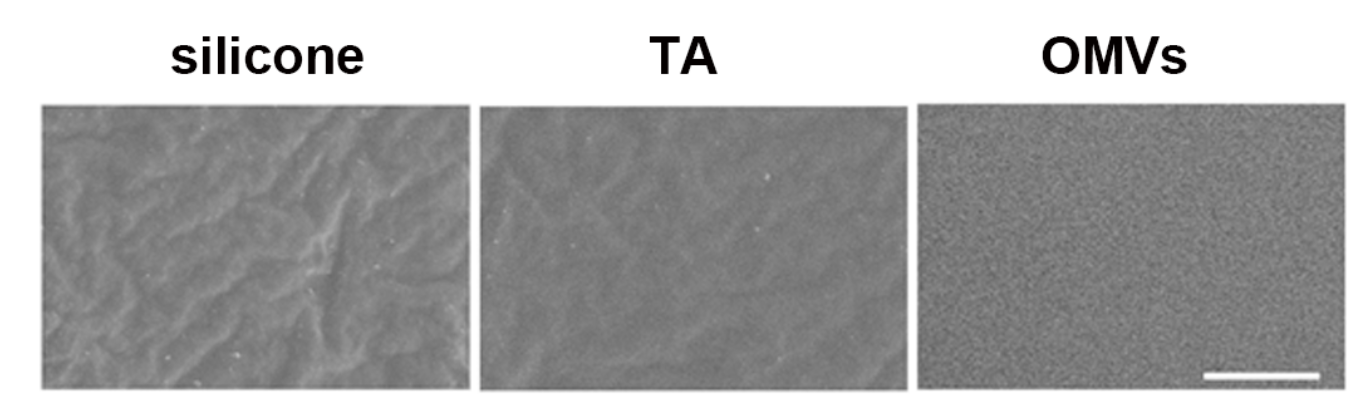
**

**Figure S2**. SEM images of silicone, silicone -TA an silicone -TA-OMVs. (scale bar:5 μm)


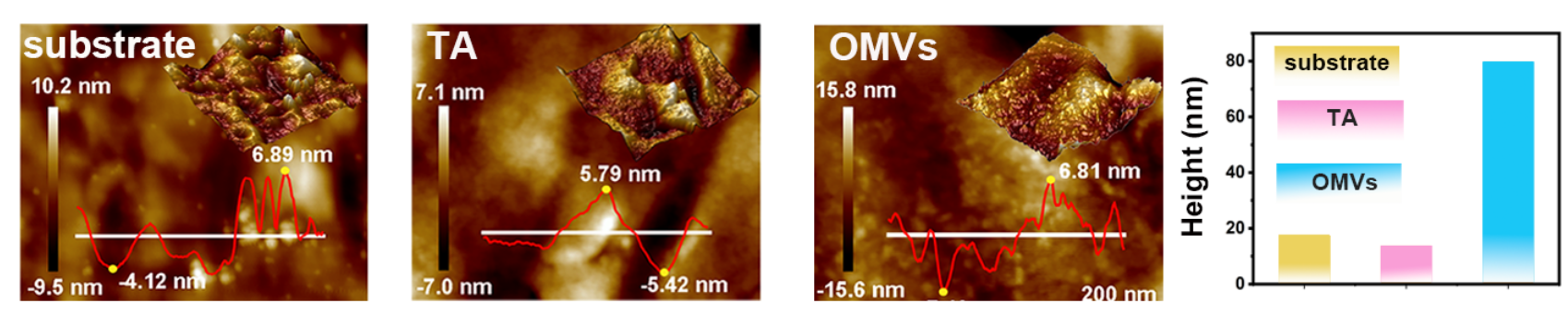


**Figure S3**. AFM images of Substrate (Ti), TA and OMVs.


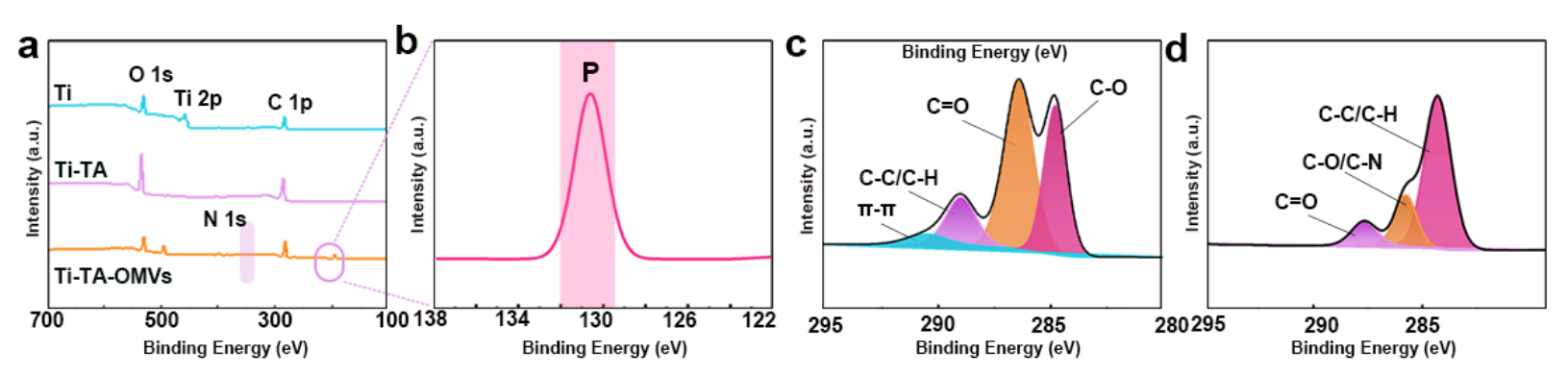


**Figure S4**. (A) X-ray Photoelectron Spectroscopy (XPS) spectra of Substrate (Ti), TA and OMVs. Core level spectra of (B) P 1s of OMVs, (C) C 1s of TA and (D) C 1s of OMVs.


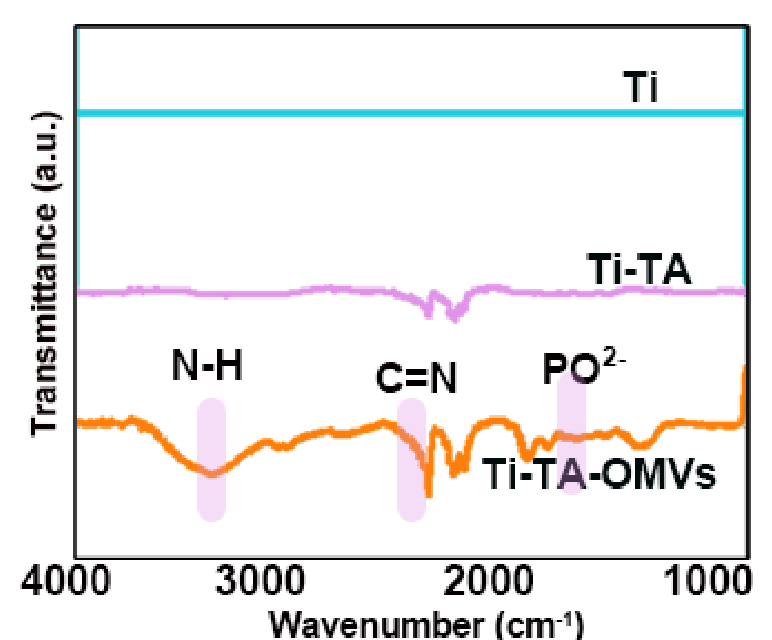


**Figure S5.** Fourier transform infrared spectrometer (FTIR) spectra of Substrate (Ti), TA and OMVs.


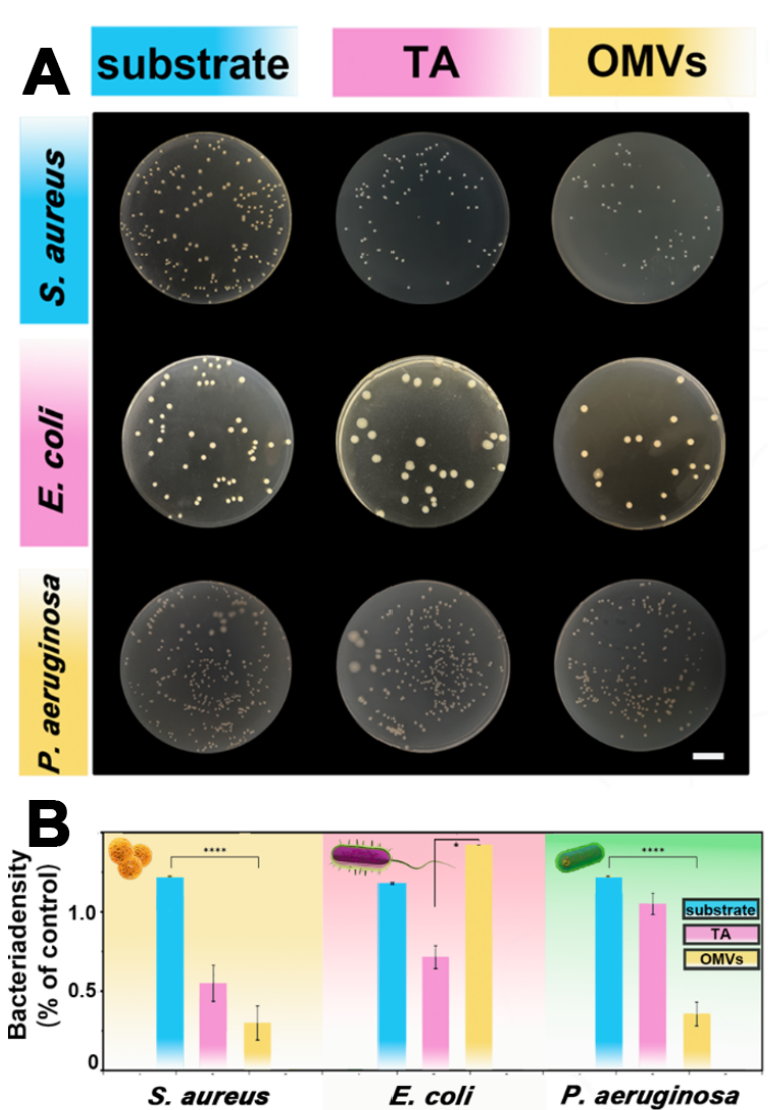


**Figure S6**. (A) Bacterial colonies treated with Substrate, TA and OMVs. (scale bar: 2 cm). (B) Bacterial density of *S. aureus*, *E. coli* and *P. aeruginosa* treated with Substrate (Ti), TA and OMVs. The data are shown as the mean ± standard deviation (n = 3).

**
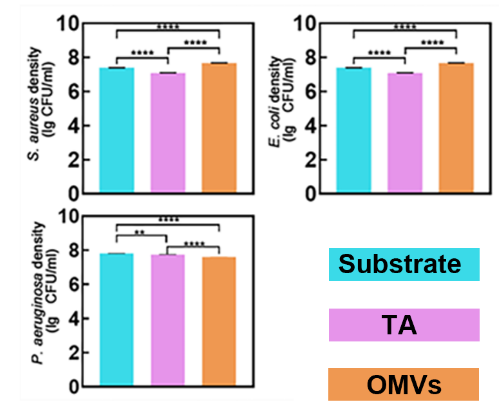
**

**Figure S7**. Bacterial density of *S. aureus, E. coli* and *P. aeruginosa* treated with different samples (Substrate (Ti), TA, OMVs). Data are presented as mean ± SD (n = 3) and analyzed using a one-way ANOVA, ***p* < 0.01, ****p* < 0.001, ns, no significance.


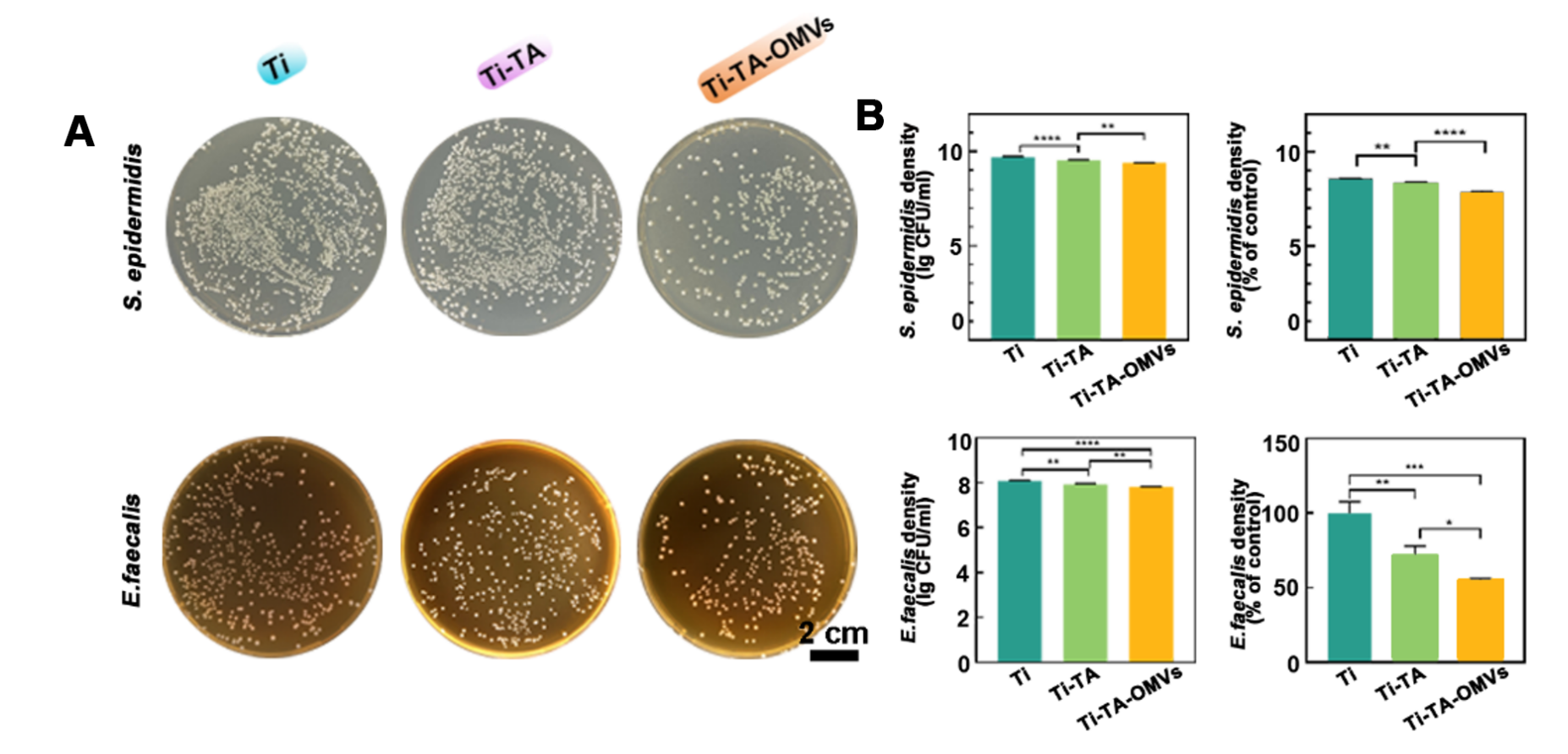


**Figure S8**. (A) Bacterial colonies treated with Substrate (Ti), TA, OMVs. (B) Bacterial density of *S. epidermidis* and *E. faecalis* treated with different samples, the data are shown as the mean ± standard deviation (n = 3).


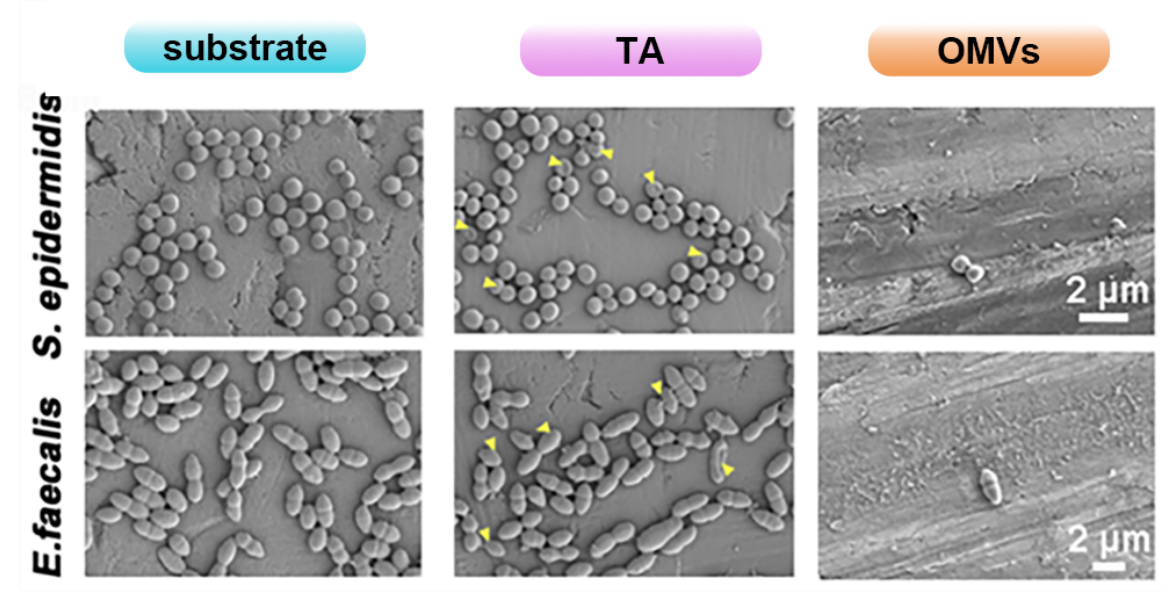


**Figure S9**. SEM images of *S. epidermidis* and *E. faecalis* cultured with Substrate (Ti), TA, OMVs.


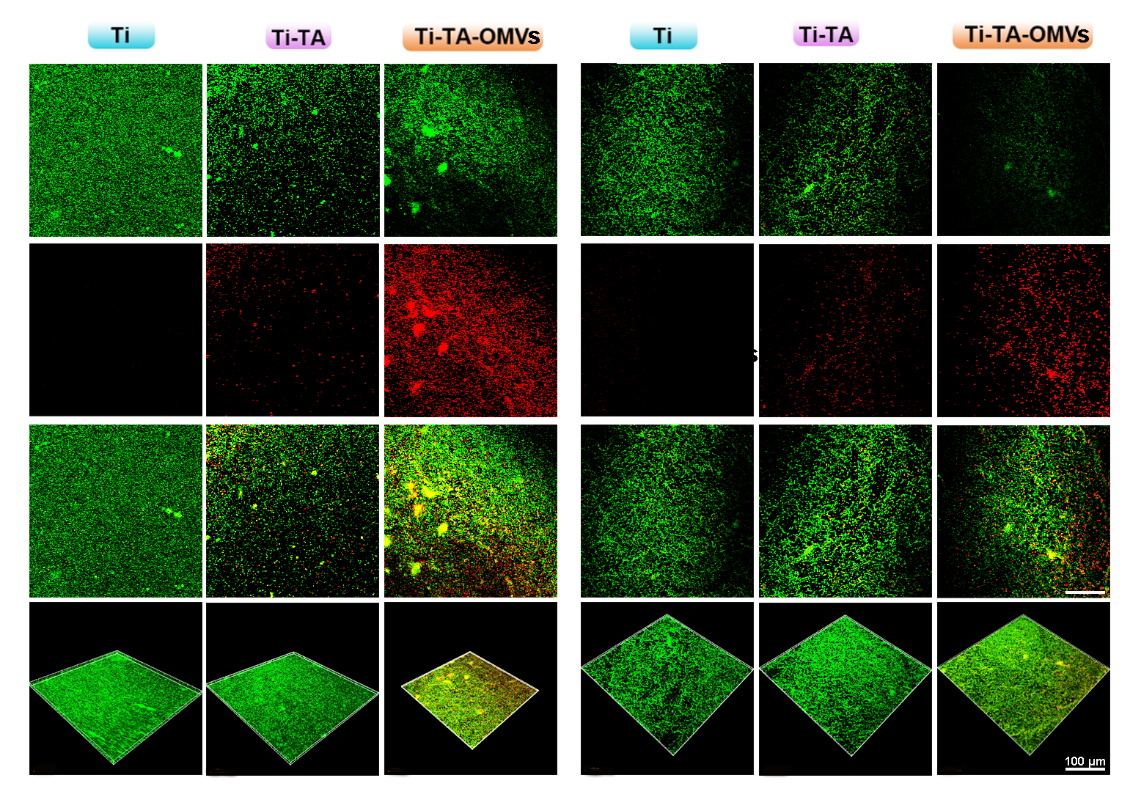


**Figure S10**. LIVE/DEAD-stained confocal images of *S. epidermidis* and *E. faecalis* cultured with Substrate (Ti), TA, OMVs.


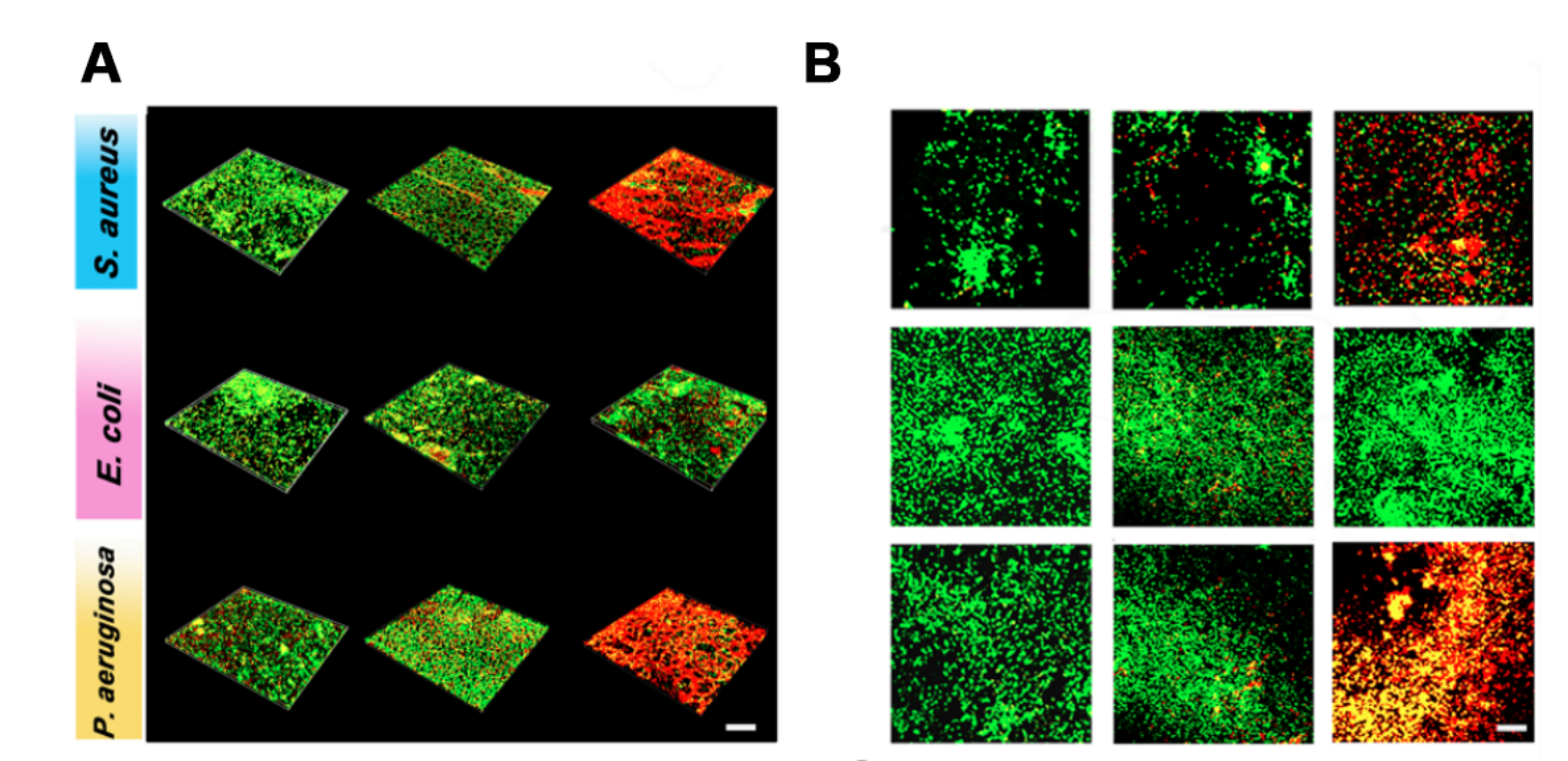


**Figure S11**.LIVE/DEAD-stained confocal images of *S. aureus*, *E. coli* and *P. aeruginosa* treated with Substrate (Ti), TA, OMVs.


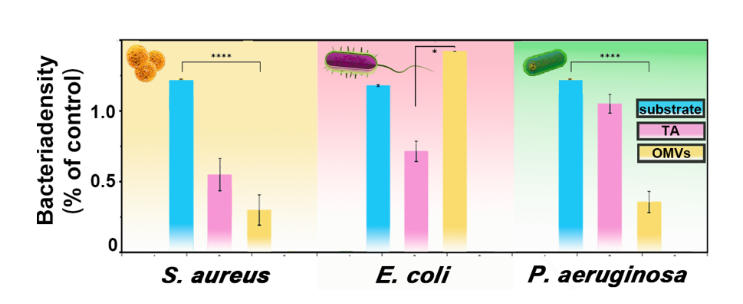


**Figure S12.** Quantitative analysis of fluorescence intensity of Substrate (Ti), TA, OMVs.


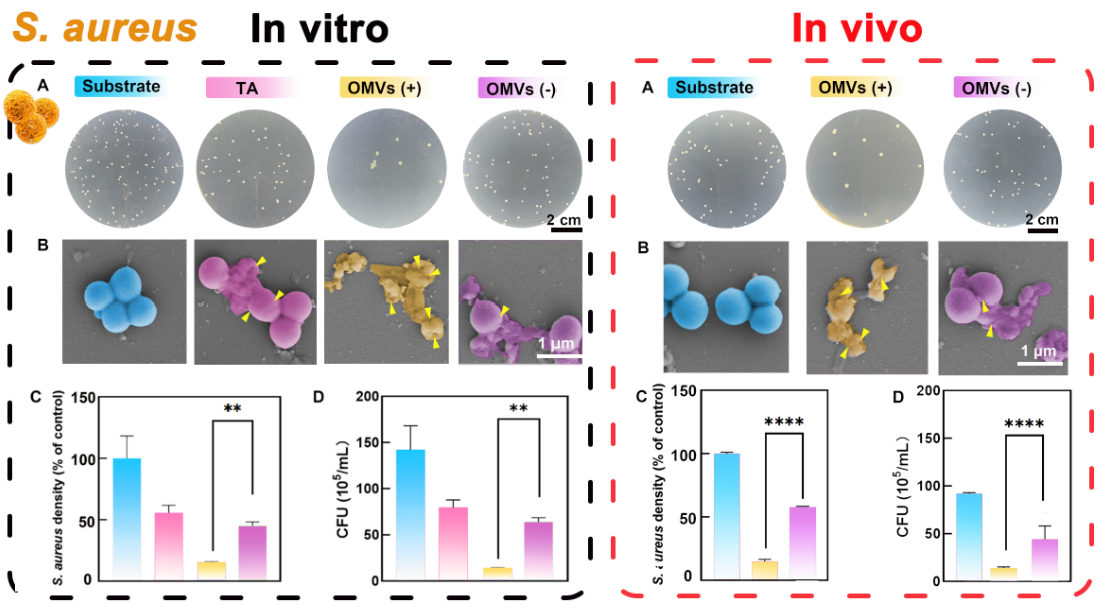


**Figure S13. Black frame**：(A) Images of bacterial colonies treated with each group in vitro. (B) SEM images of *S. aureus*. (C) and (D) Bacteriostatic ratio of *S. aureus* from spread plate results in vitro.

**Red frame：**(A) Images of bacterial colonies treated with each group in vivo. (B) SEM images of *S. aureus.* (C) and (D) Bacteriostatic ratio of *S. aureus* from spread plate results in vivo.


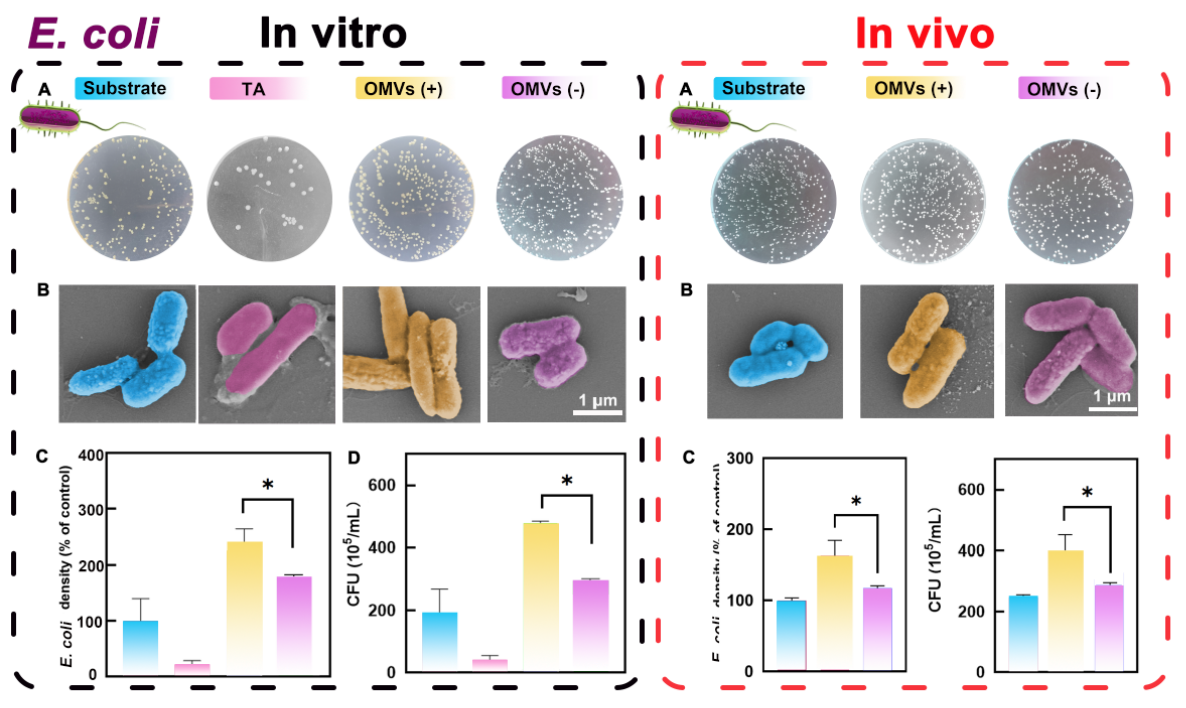


**Figure S14.Black frame**：Images of bacterial colonies treated with each group in vitro. (B) SEM images of *E. coli*. (C) and (D) Bacteriostatic ratio of *E. coli* from spread plate results in vitro.

**Red frame：**(A) Images of bacterial colonies treated with each group in vivo. (B) SEM images of *E. coli.* (C) and (D) Bacteriostatic ratio of *E. coli* from spread plate results in vivo.


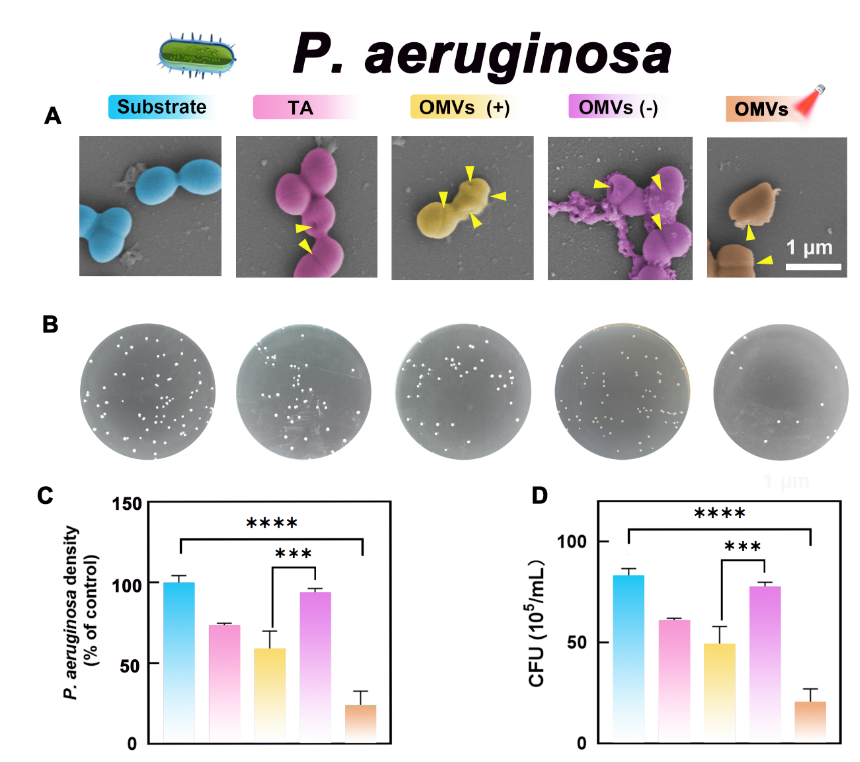


**Figure S15.** (A) Images of bacterial colonies treated with each group in vitro. (B) SEM images of *P. aeruginosa*. (C) and (D) Bacteriostatic ratio of *P. aeruginosa* from spread plate results in vitro.


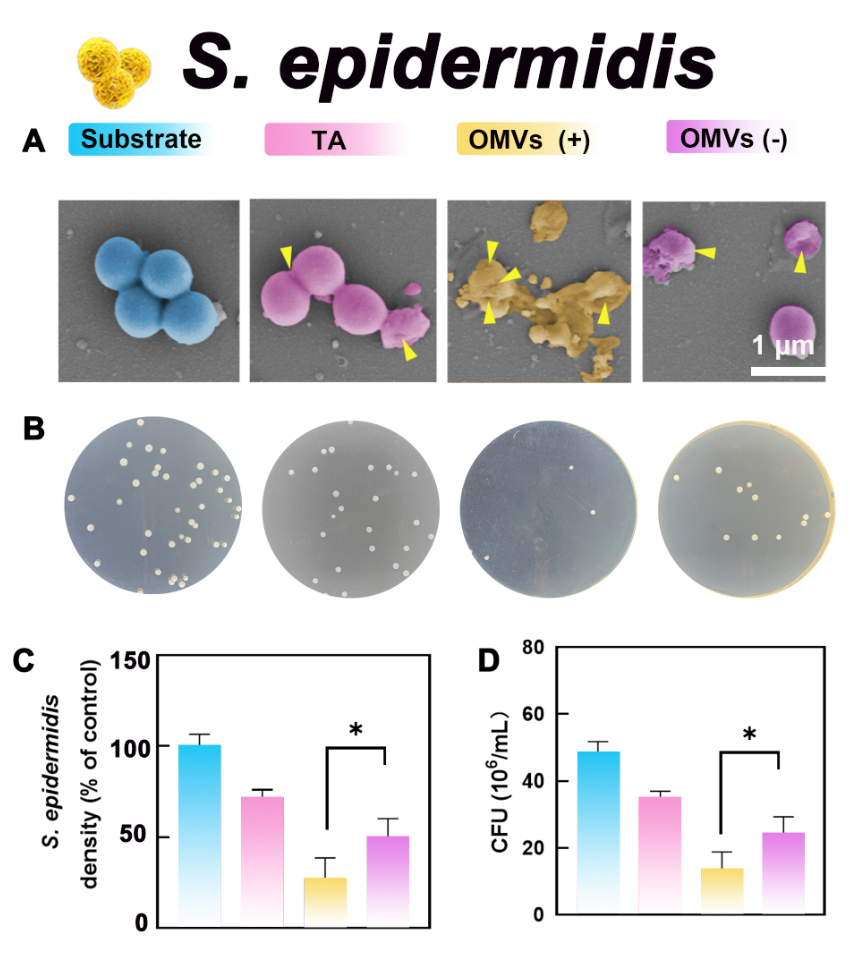


**Figure S16.** (A) Images of bacterial colonies treated with each group in vitro. (B) SEM images of *S. epidermidis*. (C) and (D) Bacteriostatic ratio of *S. epidermidis* from spread plate results in vitro.

**
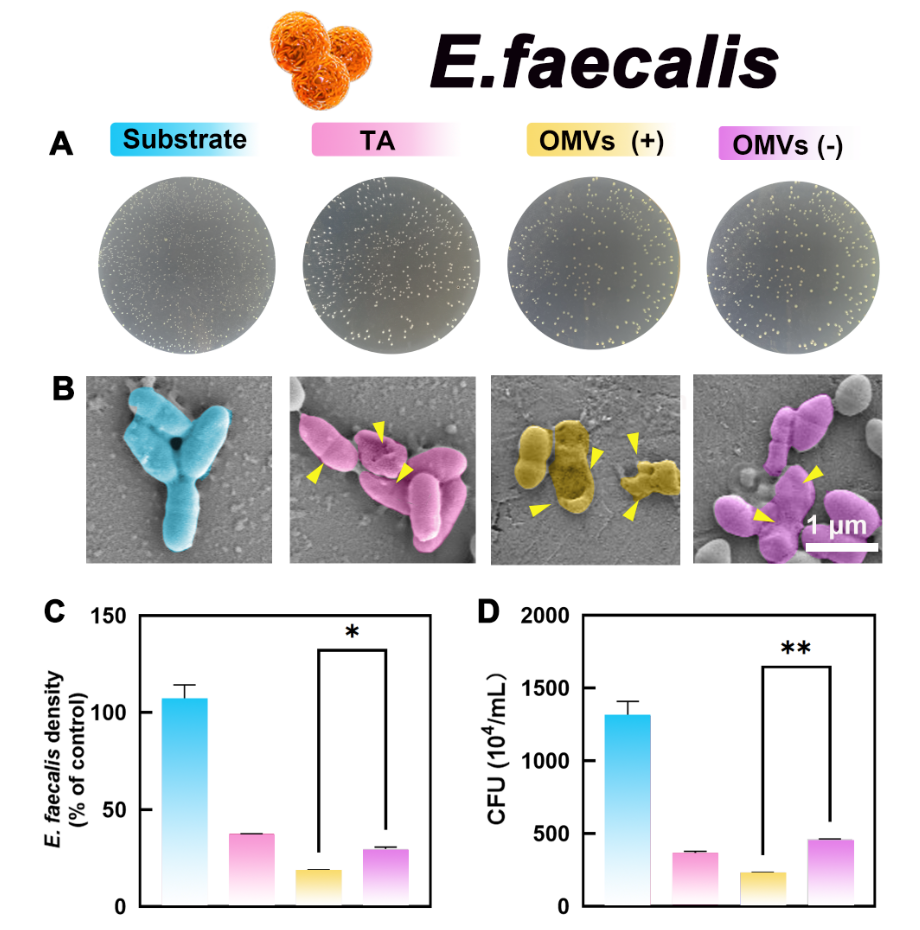
**

**Figure S17.** (A)Images of bacterial colonies treated with each group in vitro. (B) SEM images of *E. faecalis*. (C) and (D) Bacteriostatic ratio of *E. faecalis* from spread plate results in vitro.


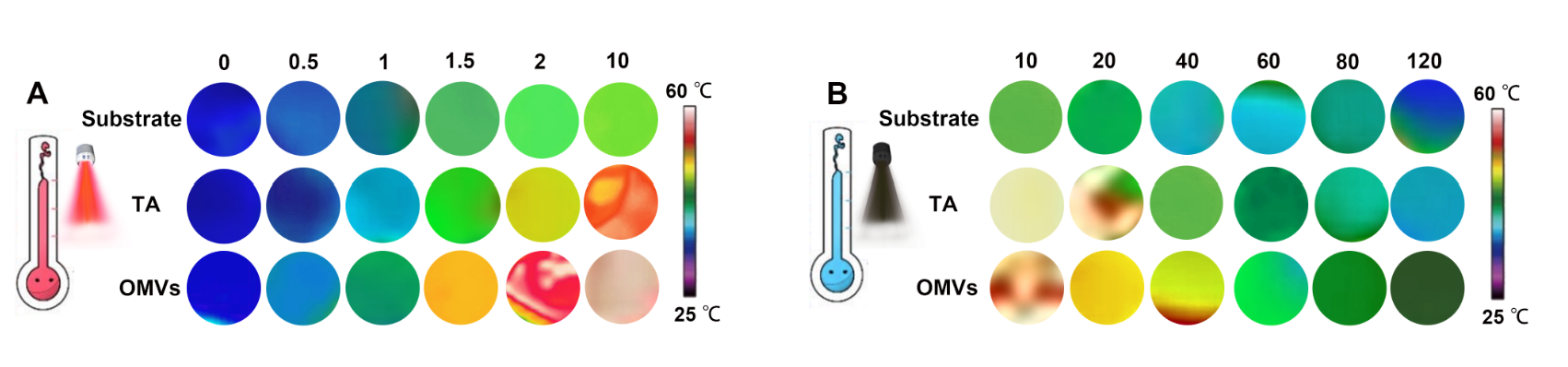


**Figure S18.** Photothermal effect evaluation. (A) Representative infrared thermal images of each group during the NIR irradiation phase; (B) Representative infrared thermal images of each group during the natural cooling phase.


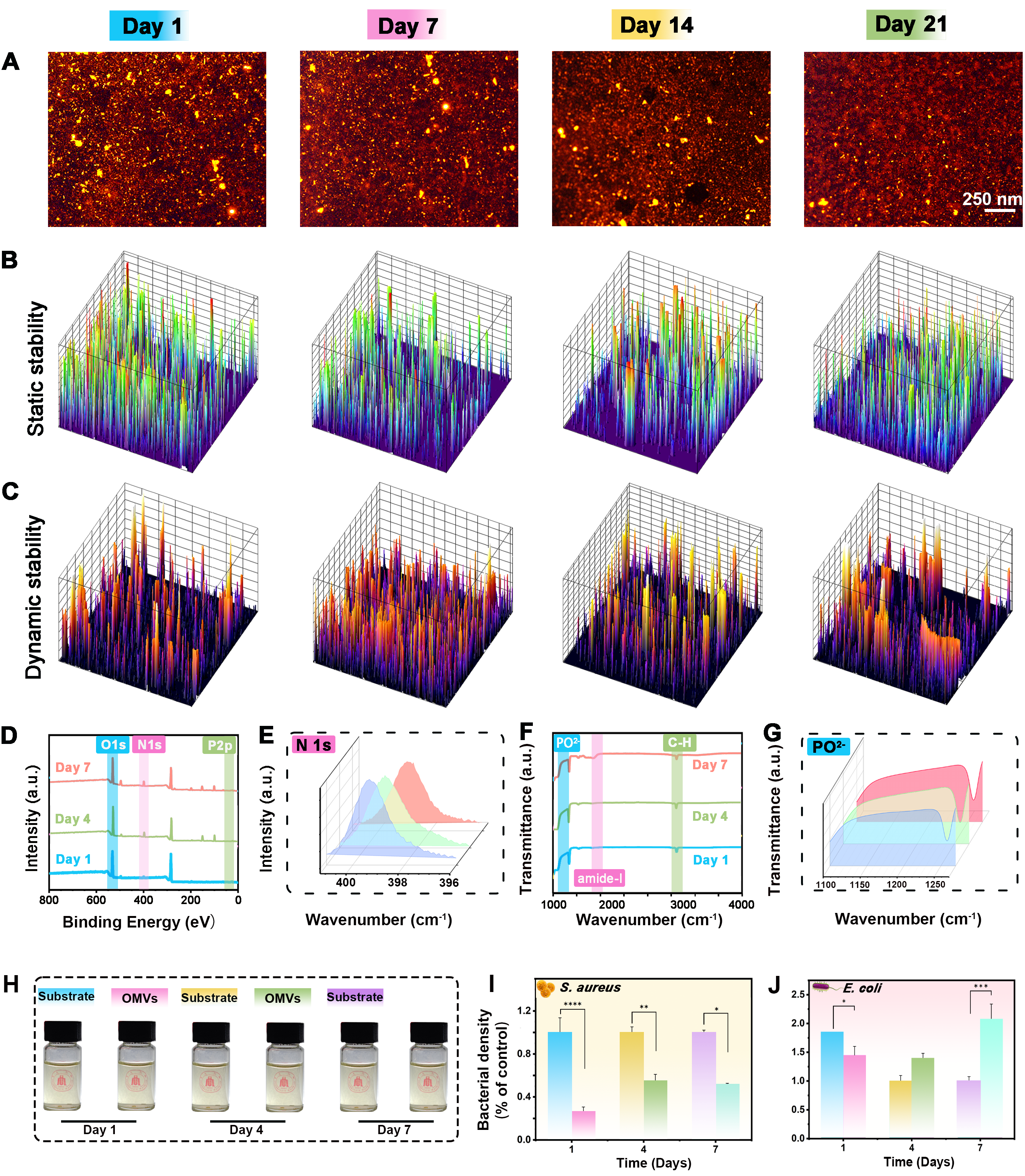


**Figure S19. Stability of the OMVs groups**. (A) Fluorescence images of OMVs were captured to assess static stability at 1, 7, 14, and 21 days. Three-dimensional fluorescence spectrogram of OMVs of (B) static stability and (C) dynamic stability. (D) XPS spectra of OMVs at 1,4 and 7 day. (E) Variation of elemental N peaks on days 1,4,7. (F) FTIR of OMVs at 1,4 and 7 day. (G) Peak changes in PO^2-^ on days 1, 4 and 7. (H) Bacterial density of *S. aureu* (I) and *E. coli* (J) treated with OMVs at different times. Data are presented as mean ± SD (n = 3) and analyzed using a one-way ANOVA, ***p* < 0.01, ****p* < 0.001, ns, no significance.


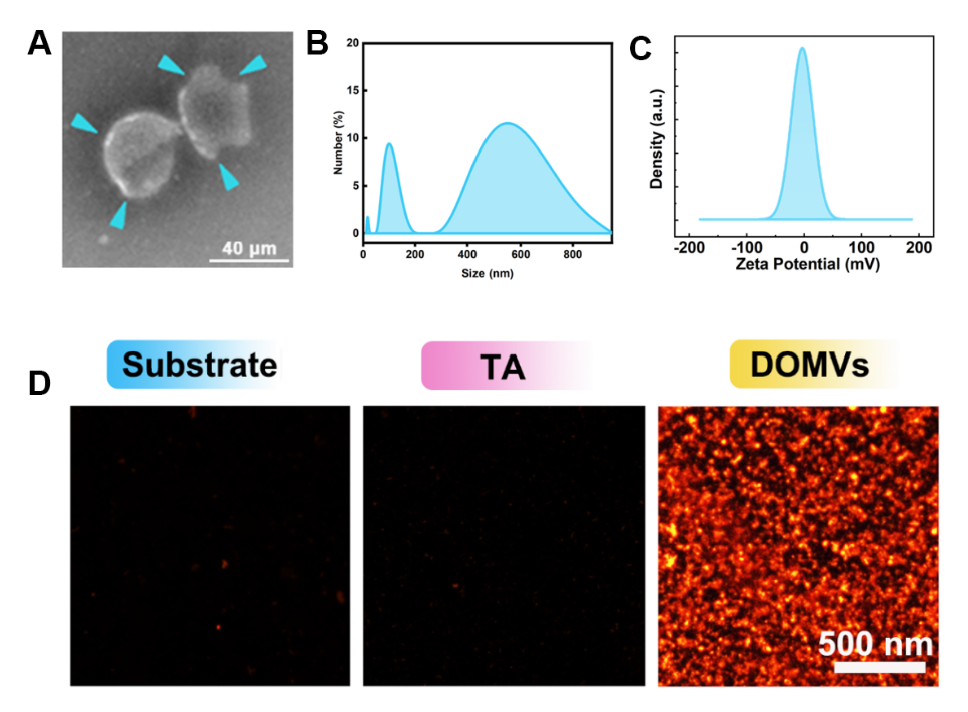


**Figure S20**. **Characterization of DOMVs and DOMVs coating**. (A) TEM images, and (B) diameter of Destroyed OMVs. (C) Zeta potential (D) DiD-stained of Substrate, TA and OMVs.


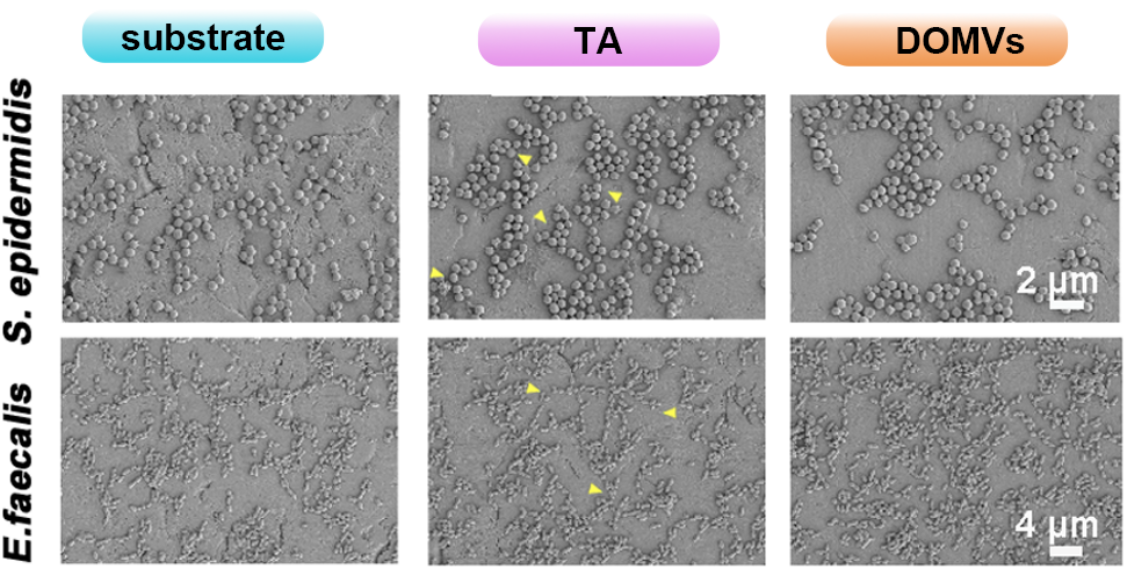


**Figure S21**. SEM images of *E. faecalis* and *S. epidermidis* cultured with Substrate, TA, DOMVs.


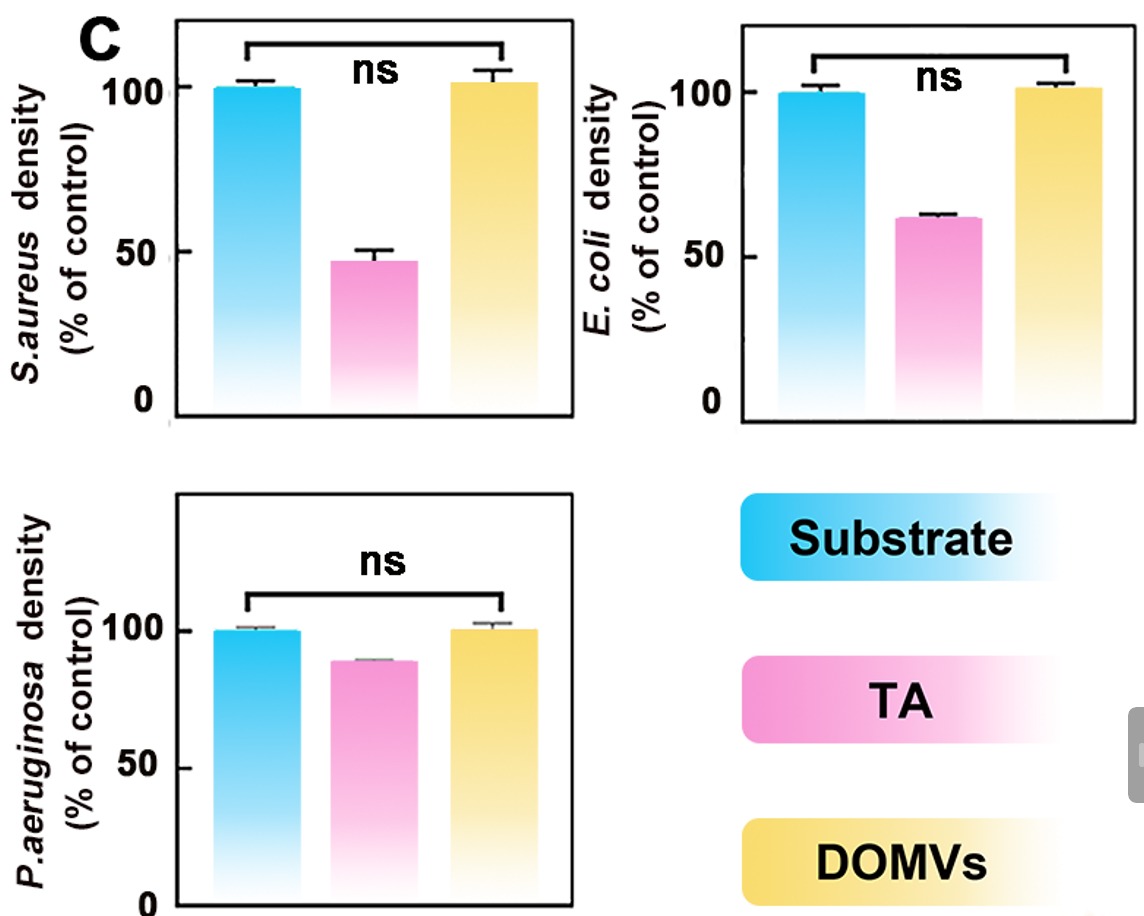


**Figure S22.** Bacterial density of *S. aureus, E. coli* and *P. aeruginosa* treated with Substrate, TA and DOMVs. Data are presented as mean ± SD (n = 3) and analyzed using a one-way ANOVA, ***p* < 0.01, ****p* < 0.001, ns, no significance.

**
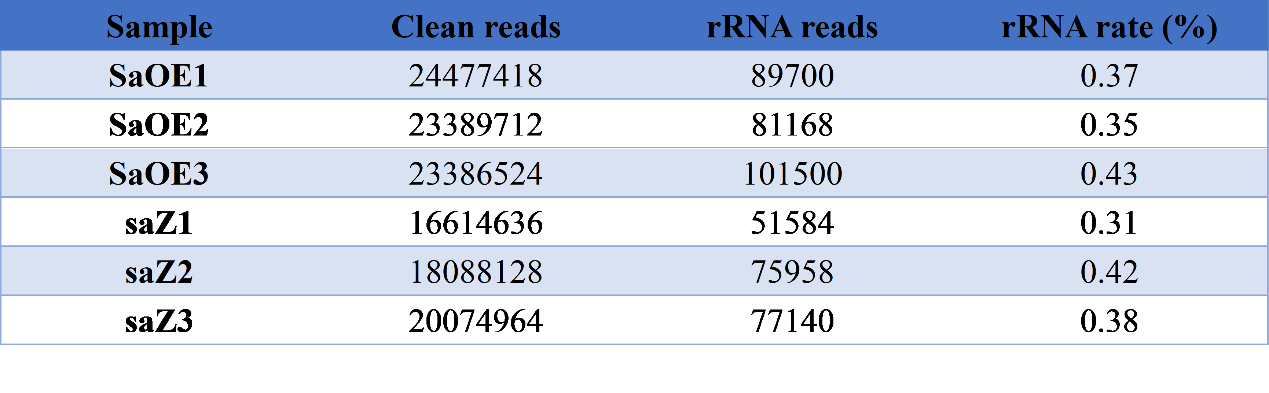
**

**Figure S23.** Ribosome contamination assessment of *S. aureus* exposed to OMVs. The reads obtained after QC were compared with the Rfam database (BLAST+ 2.7.1, comparison criteria: E-value not greater than 1^e-5^) and reads matching ribosomal RNA (rRNA) were removed.


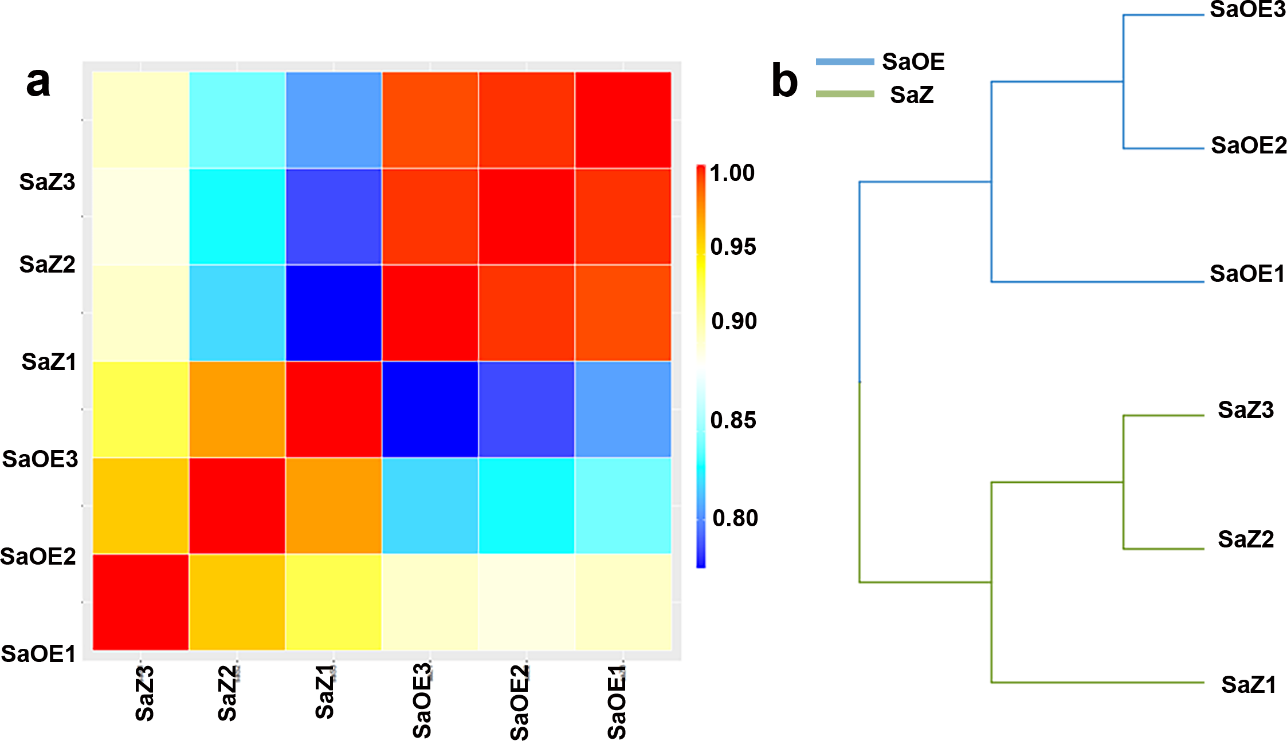


**Figure S24.** Sample correlation analyses of *S. aureus* exposed to OMVs (A). Correlation coefficients were calculated for each sample based on gene expression levels, calculated using pearson's method and presented in the form of heat maps. The horizontal and vertical coordinates in the graph are the names of the samples, and the colour blocks at different positions represent the magnitude of correlation coefficients between the samples at the corresponding positions, with red indicating positive correlation and blue indicating negative correlation. Sample cluster analysis of *S. aureus* exposed to OMVs (B). Based on the gene expression of each sample, the Pearson Correlation Efficiency (PCE) was calculated to indicate the similarity between two samples. Then the samples with high similarity were grouped into one category using Hierarchical Cluster, and so on, to get the overall clustering results of the samples.


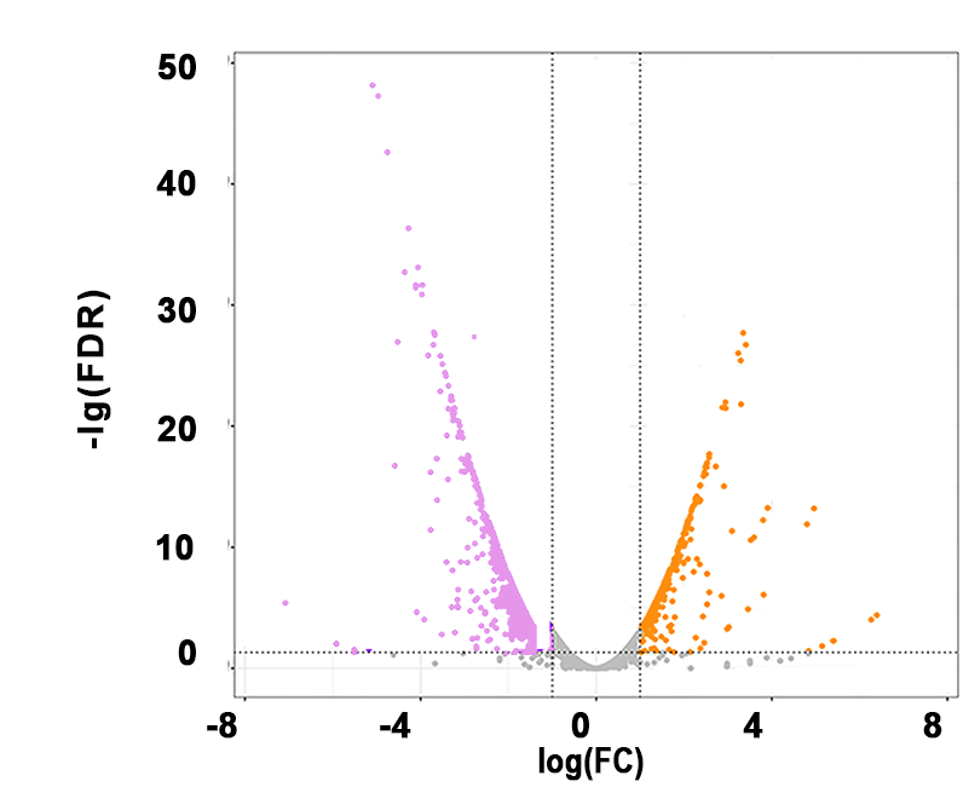


**Figure S25.** Volcano map for the distribution of DEGs.


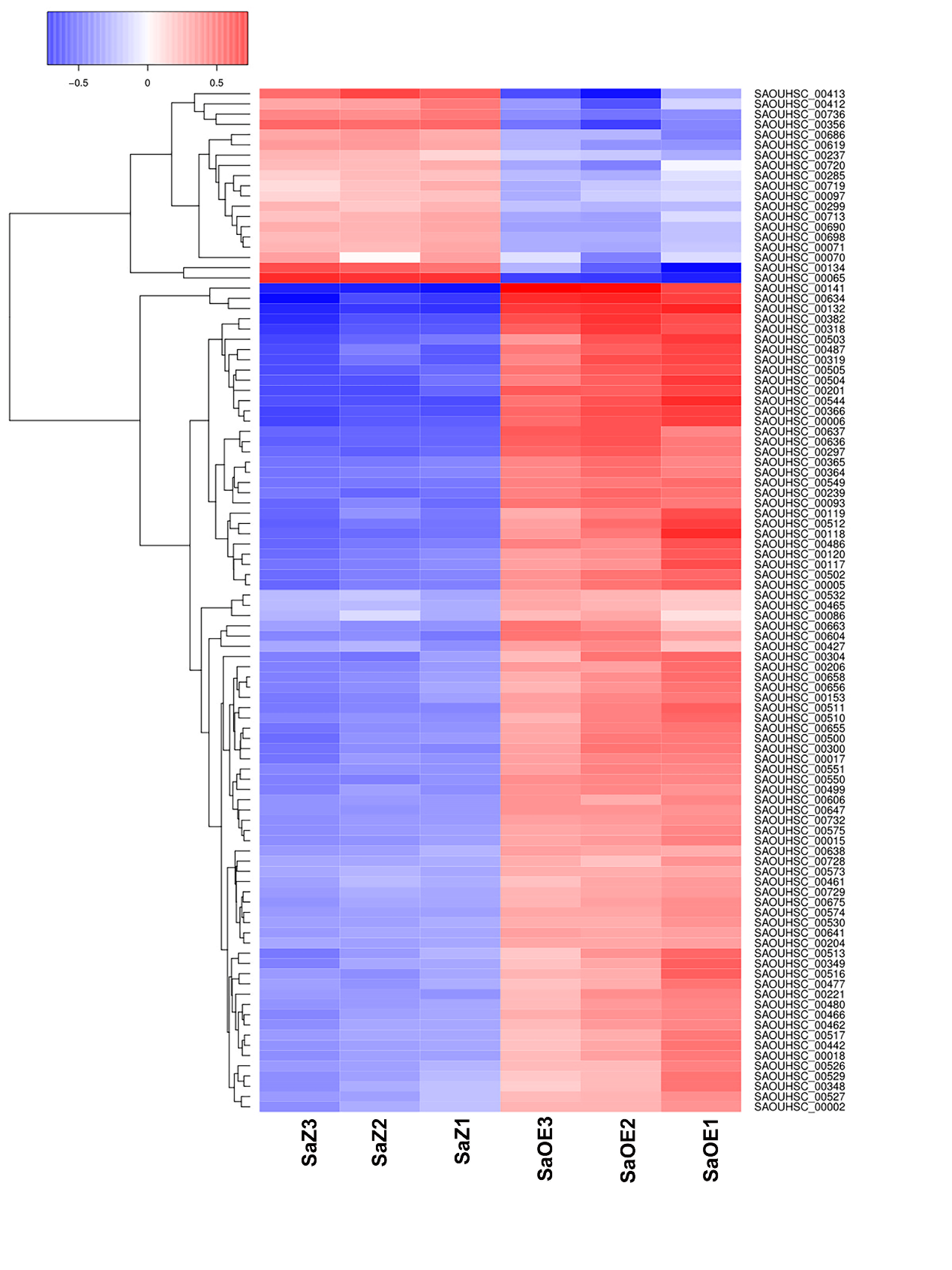


**Figure S26.** Heat map of DEGs **o**f *E. coli exposed* to *S. aureus***.** Each column in the graph represents a sample, each row represents a gene, the color in the graph indicates the expression size of the gene in the group of samples (log10 FPKM), red means that the gene has a higher expression in the sample, and blue means that it has a lower expression, and the specific trend of the expression size changes can be seen in the numbers under the color bar on the upper left. The left side is the dendrogram of gene clustering, the right side is the name of the genes, the closer the branches of two genes are, the closer their expressions are the bottom side is the name of the samples.


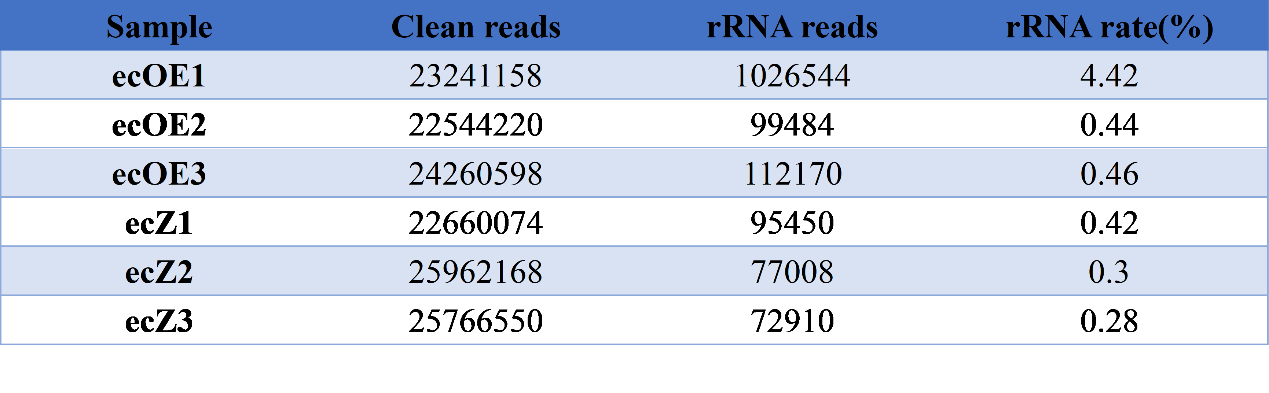


**Figure S27.** Ribosome contamination assessment of *E. coli* exposed to OMVs. The reads obtained after QC were compared with the Rfam database (BLAST+ 2.7.1, comparison criteria: E-value not greater than 1^e-5^) and reads matching ribosomal RNA (rRNA) were removed.

**
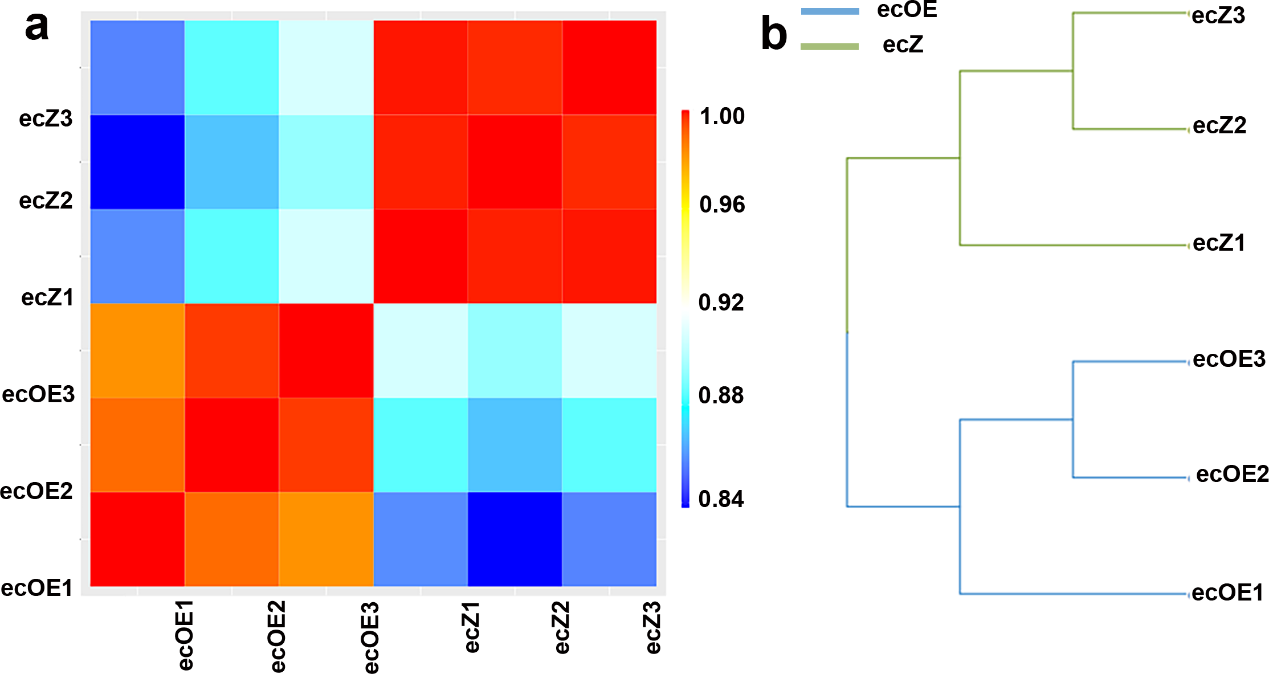
**

**Figure S28.** Sample correlation analyses of *E. coli* exposed to OMVs (A). Correlation coefficients were calculated for each sample based on gene expression levels, calculated using pearson's method and presented in the form of heat maps. The horizontal and vertical coordinates in the graph are the names of the samples, and the colour blocks at different positions represent the magnitude of correlation coefficients between the samples at the corresponding positions, with red indicating positive correlation and blue indicating negative correlation. Sample cluster analysis of *E. coli exposed* to OMVs (B). Based on the gene expression of each sample, the Pearson Correlation Efficiency (PCE) was calculated to indicate the similarity between two samples. Then the samples with high similarity were grouped into one category using Hierarchical Cluster, and so on, to get the overall clustering results of the samples.


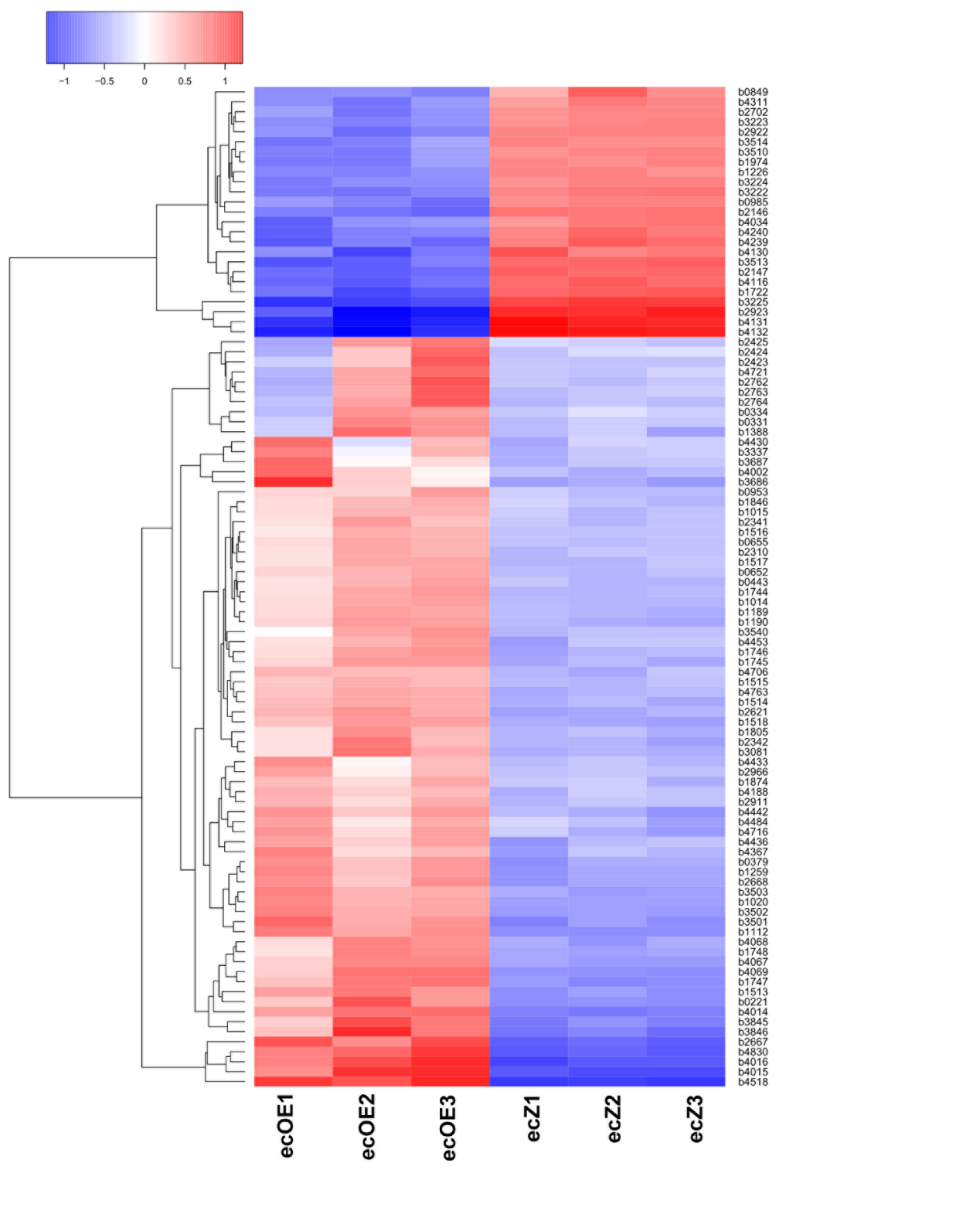


**Figure S29.** Heat map of DEGs of *E. coli exposed* to OMVs. Each column in the graph represents a sample, each row represents a gene, the color in the graph indicates the expression size of the gene in the group of samples (log10 FPKM), red means that the gene has a higher expression in the sample, and blue means that it has a lower expression, and the specific trend of the expression size changes can be seen in the numbers under the color bar on the upper left. The left side is the dendrogram of gene clustering, the right side is the name of the genes, the closer the branches of two genes are, the closer their expressions are the bottom side is the name of the samples.


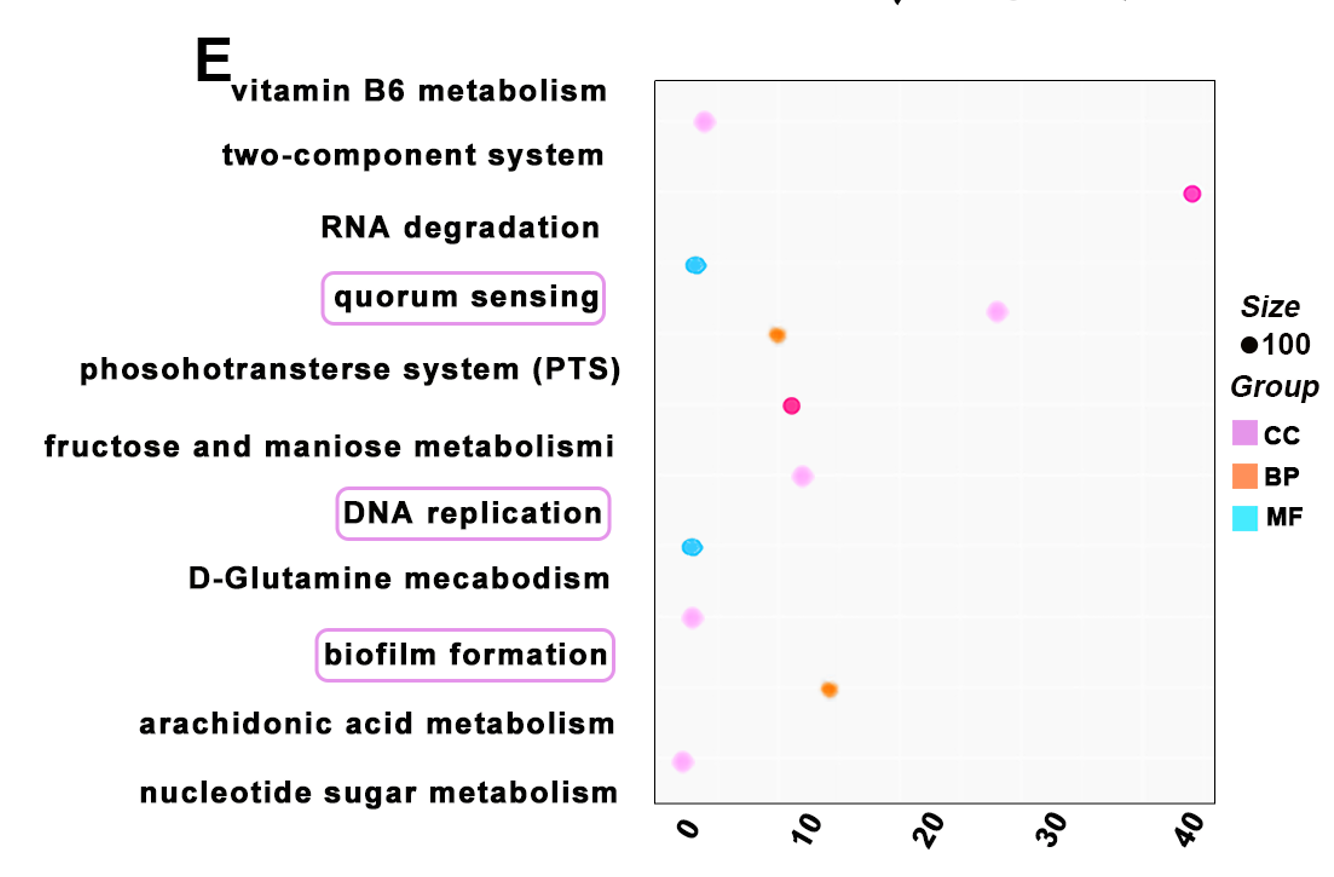


**Figure S30.** Downregulated DEGs enriched in the KEGG pathway.


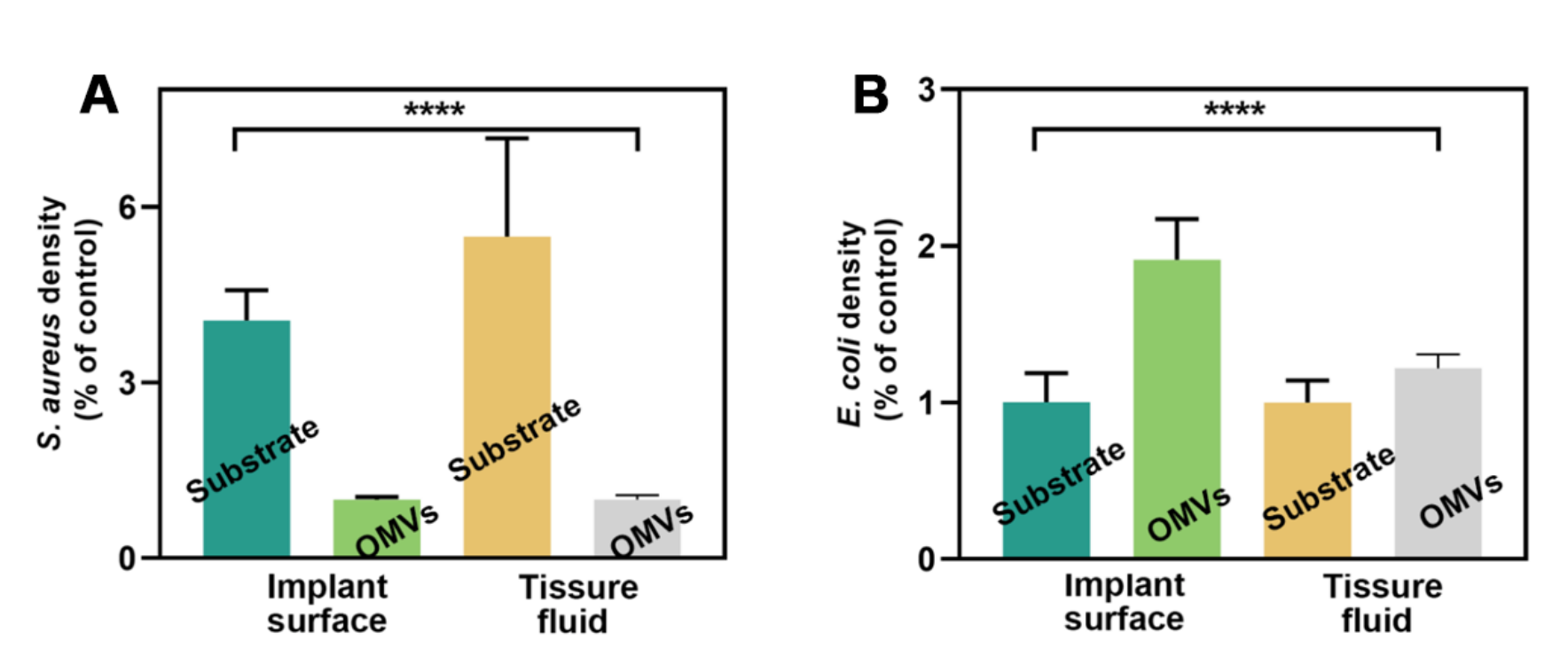


**Figure S31.** (A) Bacterial density of *S. aureus* remaining in Ti based implant surface and exudate. (B) Bacterial density of *E. coli* remaining in Ti based implant surface and exudate. The data are shown as the mean ± standard deviation (n = 3).


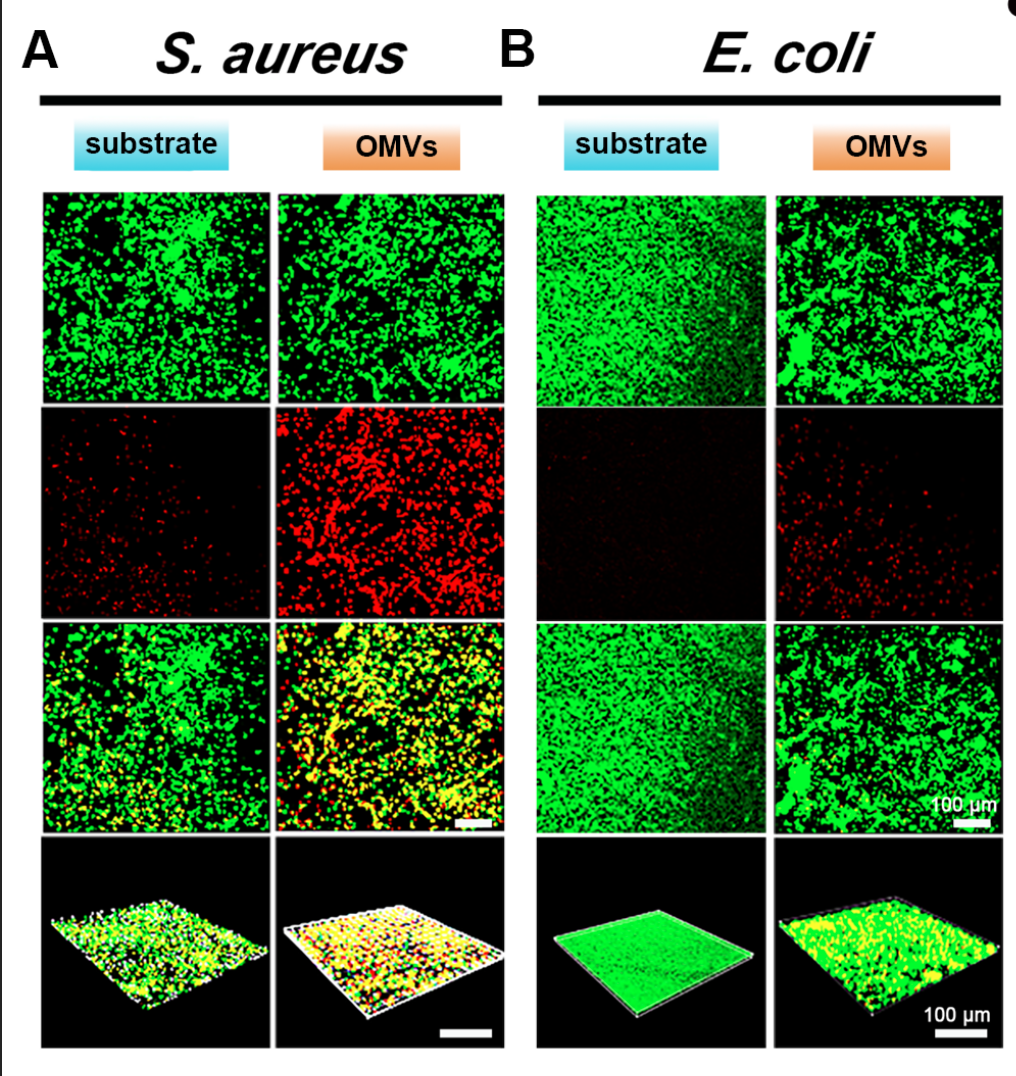


**Figure S32.** LIVE/DEAD stained confocal images of *S. aureus* (A) and *E. coli* (B) on the surface of titanium Substrate and OMVs retrieved from rats.


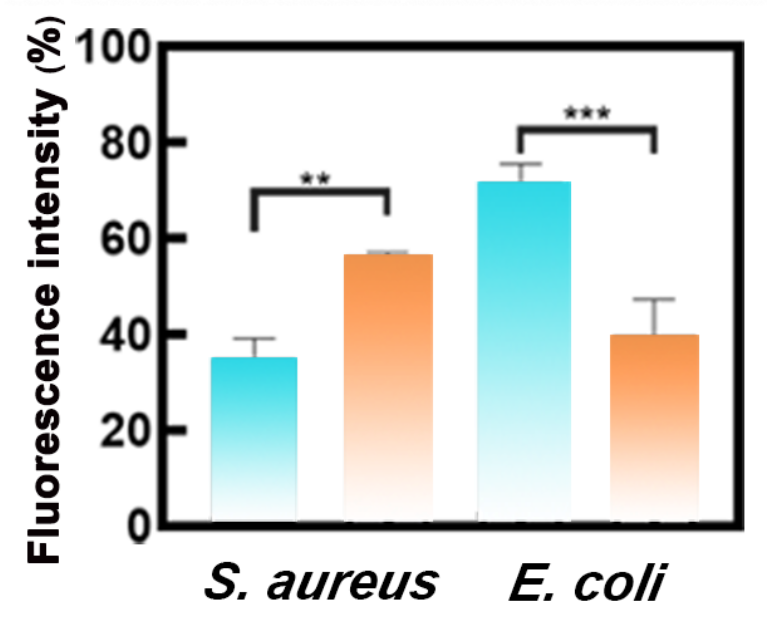
**Figure S33.** Quantitative red fluorescence analysis of titanium implant surface.


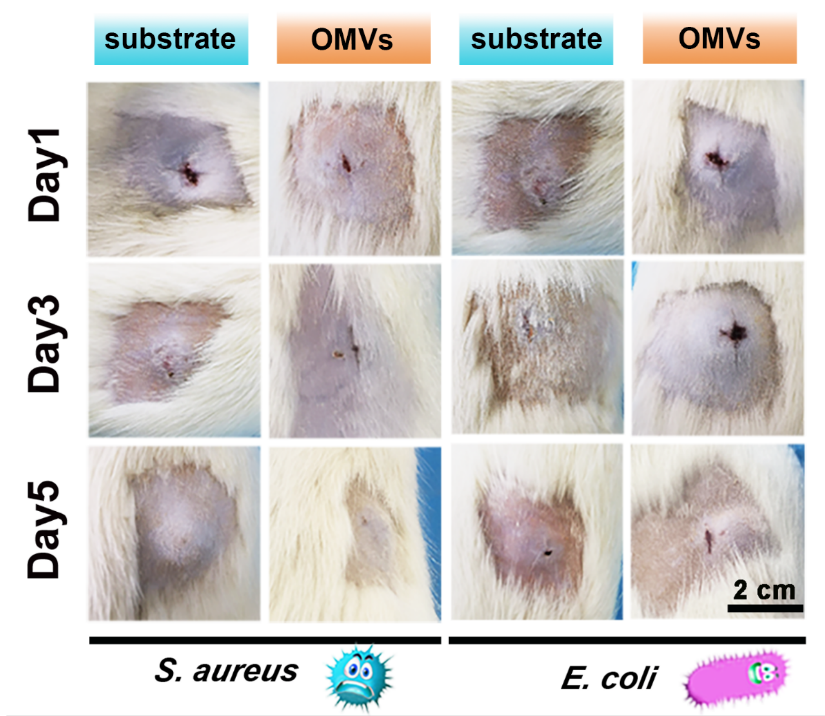


**Figure S34.** Pictures of the healing process in infected wounds of titanium implant models.

**
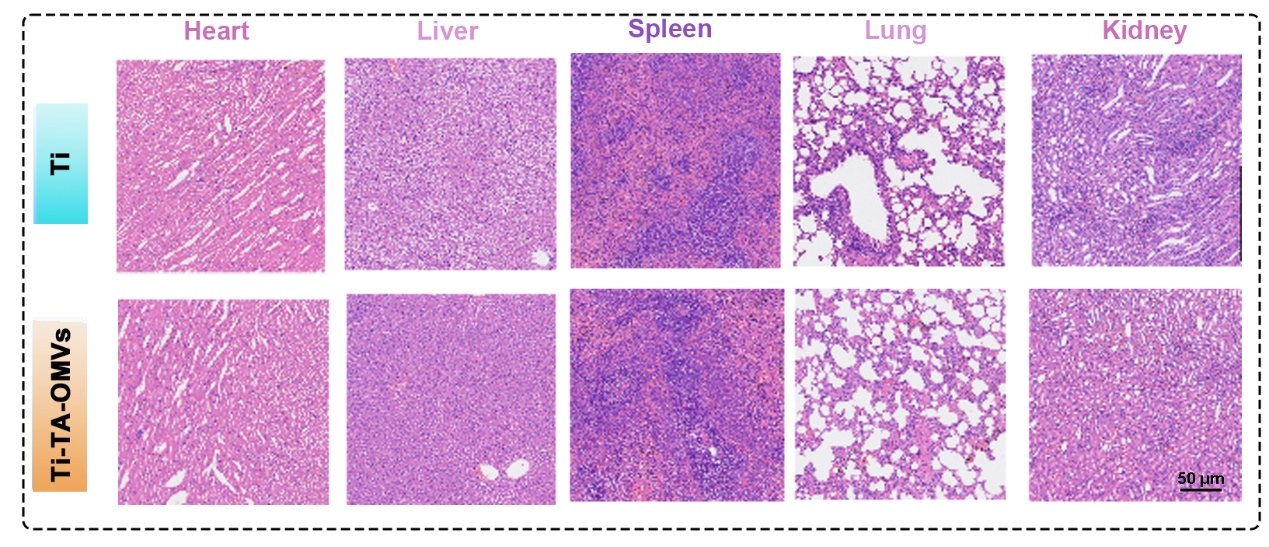
**

**Figure S35**. Biotoxicity analysis: H&E staining results of major organs in *S. aureus* infected rats after different treatments.


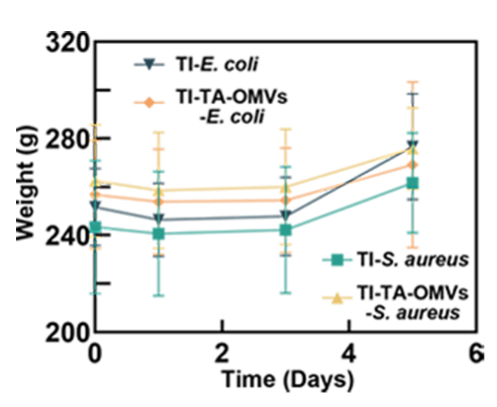


**Figure S36**. Biotoxicity analysis: changes of body weight in different groups of titanium implant models.


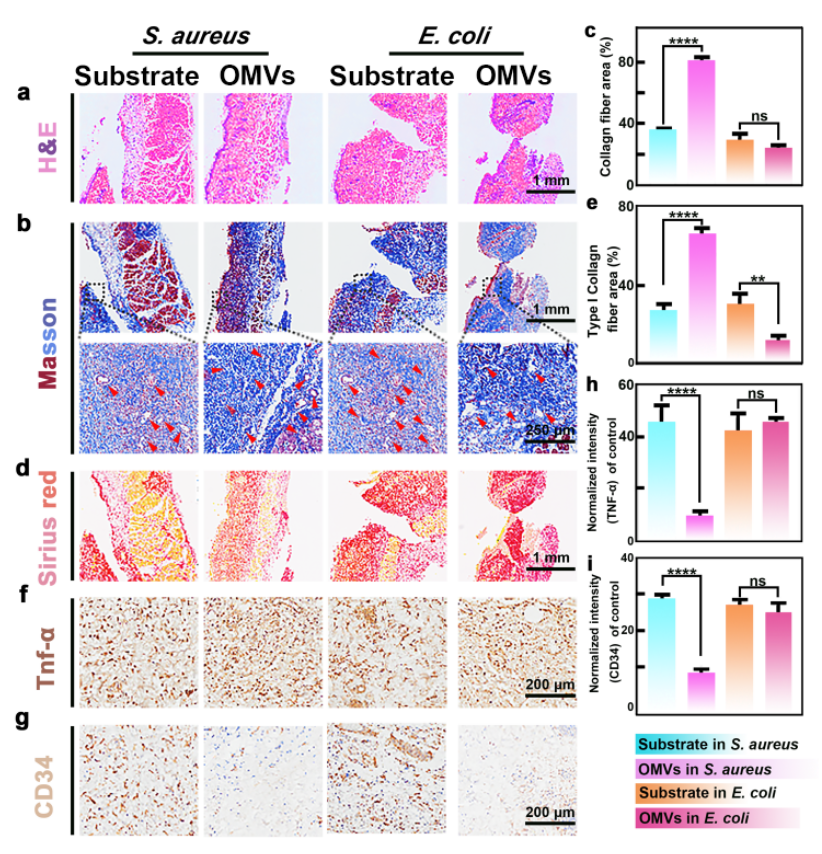


**Figure S37**. Histopathologic analysis of slices of the titanium substrate model.(A) H&E staining results of skin tissues after different treatments. (B) Masson’s trichrome staining results (red arrows represent the new vessels) and (C) the corresponding quantitative collagen area fraction data. (D) Sirius red staining images of the epidermal histological sections and (E) the corresponding quantitative type I collagen area fraction data. Representative immunohistochemistry images for (F) cluster of differentiation 34 (CD34) and (G) tumor necrosis factor α (TNF-α). The relevant quantitative analysis of (H) TNF-α and (I) CD34 positive signals. The data are shown as the mean ± standard deviation (n = 3)ssss


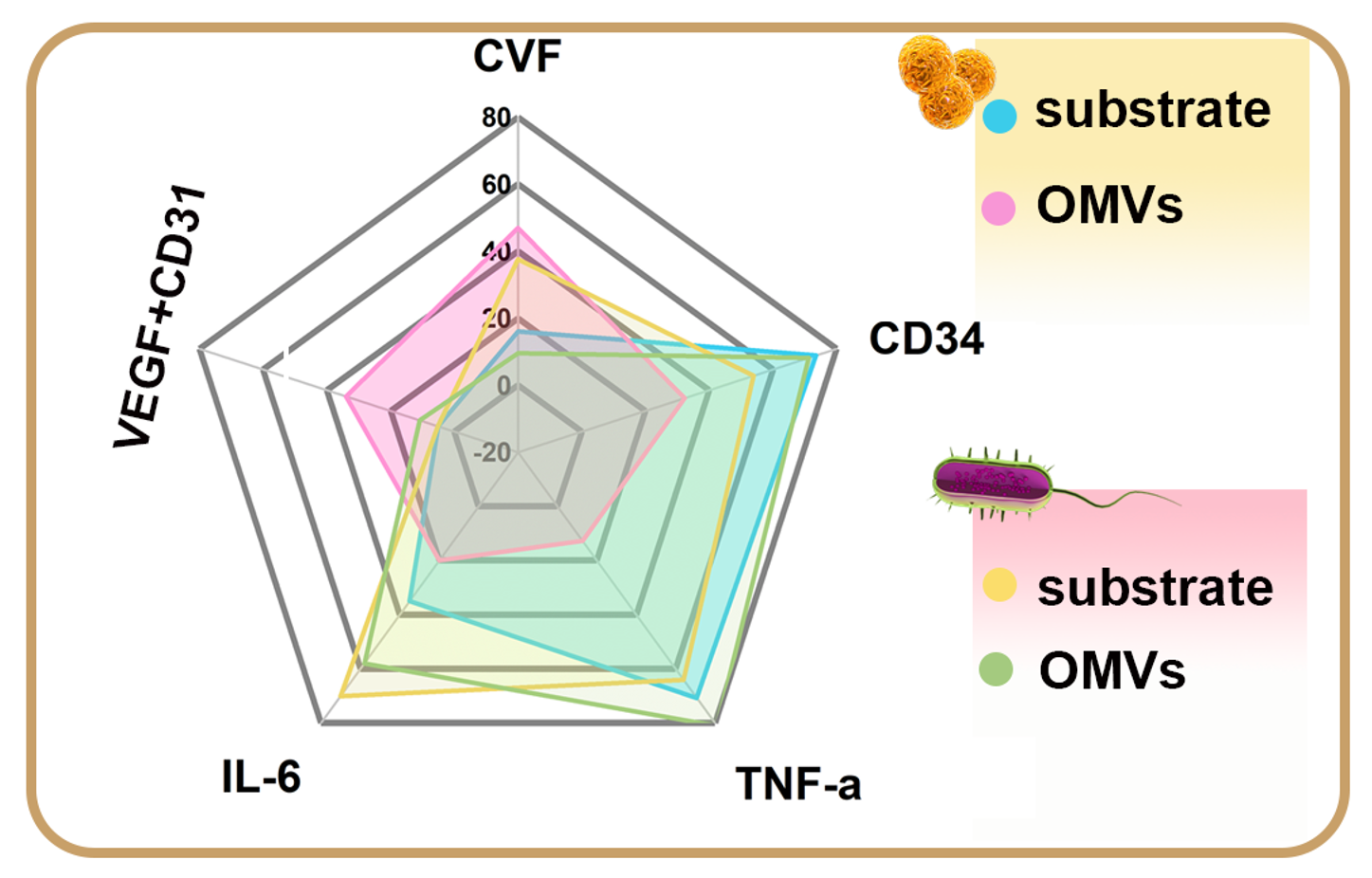


**Figure S38.** Comprehensive performance of Substrate, TA, OMVs.


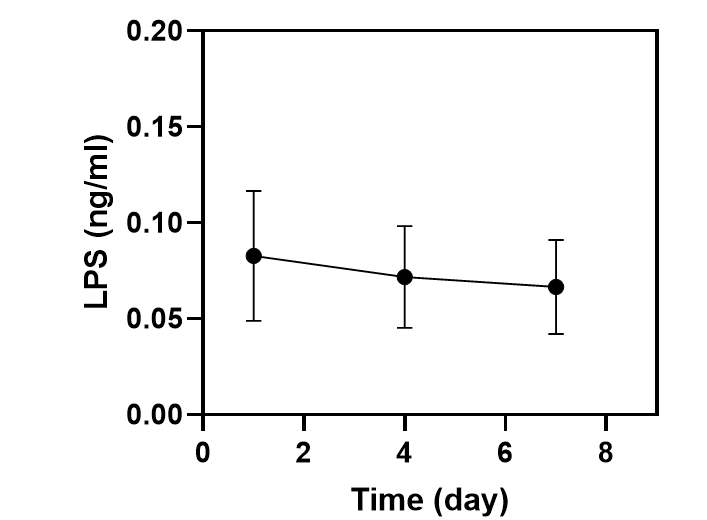


**Figure S39** LPS content release on days 1, 4 and 7. (n=3)
